# Predicting Crystallization Propensity of Proteins from *Arabidopsis Thaliana*

**DOI:** 10.1186/s12575-015-0029-3

**Published:** 2015-11-23

**Authors:** Shaomin Yan, Guang Wu

**Affiliations:** State Key Laboratory of Non-food Biomass Enzyme Technology, National Engineering Research Center for Non-food Biorefinery, Guangxi Key Laboratory of Biorefinery, Guangxi Academy of Sciences, 98 Daling Road, Nanning, Guangxi 530007 China

**Keywords:** Amino acid characteristics, *Arabidopsis thaliana*, Crystallization propensity, Modeling, Protein

## Abstract

**Background:**

Many studies have correlated characteristics of amino acids with crystallization propensity, as part of the effort to determine the factors that affect the propensity of protein crystallization. However, these characteristics are constant; that is, the encoded amino acid sequences have the same value for each type of amino acid. To overcome this inflexibility, three dynamic characteristics of amino acids and protein were introduced to analyze the crystallization propensity of proteins. Both logistic regression and neural network models were used to correlate each of two dynamic characteristics with the crystallization propensity of 301 proteins from *Arabidopsis thaliana*, and their results were compared with those obtained from each of 531 constant amino acid characteristics, which served as the benchmark.

**Results:**

The neural network model was more powerful for predicting the crystallization propensity of proteins than the logistic regression model. Compared with the benchmark, the dynamic characteristics of amino acids provided good prediction results for the crystallization propensity, and the distribution probability gave the highest sensitivity. Using 90 % accuracy as a cutoff point, the predictable portion of *A. thaliana* portions was ranked, and the statistical analysis showed that the larger the predictable portion, the better the prediction.

**Conclusions:**

These results demonstrate that dynamic characteristics have a certain relationship with the crystallization propensity, and they could be helpful for the prediction of protein crystallization, which may provide a theoretical concept for certain proteins before conducting experimental crystallization.

**Electronic supplementary material:**

The online version of this article (doi:10.1186/s12575-015-0029-3) contains supplementary material, which is available to authorized users.

## Background

Protein crystallization is truly a state-of-art technology because its success is a combination of many factors involved in the crystallization process. Huge efforts have so far been made to determine crucial factors involved in the protein crystallization process based on sequence information [1–4] in order to discover an indicator of whether a protein can be crystallized. Needless to say, this indicator should reveal the very nature of proteins in relation to their crystallization. As a result, initial attention was given to the protein length and protein isoelectric point in their correlation with protein crystallization [5]. These protein characteristics could account for the nature of protein crystallization to some degree but not all. Efforts are therefore directed to various characteristics which can represent any aspect of the nature of protein, such as physiochemical properties of amino acids [5–12], in correlation with the success rate of protein crystallization. Indeed, these characteristics are numerical values, each represent an aspect of the nature of protein, and they currently number more than 540 in the amino acid database AAIndex [13].

Some characteristics account only for a protein, such as protein length, while some characteristics account only for an amino acid, such as molecular weight of the amino acid, but there are few characteristics accounting for both together. The nature of a protein is not the sum of the natures of its composite amino acids, although a characteristic for a protein might be an addition of the characteristics of its composite amino acids—for example, the protein isoelectric point is the sum of the composite amino acid isoelectric points. Over the last decade, we have determined three characteristics of amino acids that vary in different proteins because they account for the nature of both the protein and its composed amino acids [14]. We attempted to determine whether these three characteristics could account for protein crystallization to some promising degree [15–19], although we would not expect them to account for the whole nature of the protein in relation to protein crystallization. The theoretical approach is to set a model, which is more likely to be of a regression type, to build a relationship between the protein’s and amino acids’ characteristics and the successful rate of protein crystallization [5–12, 15–19].

*Arabidopsis thaliana* is a model species broadly used in plant research, many aspects of which draw great attention such as the circadian clock genes [20], the control of key regulatory genes at many stages of development during the life cycle [21], the diversity of dual targeting mechanisms [22], B-GATA transcription factors [23], gravity influence on the growth direction of higher plants [24], substrate specificity, and multiple stress tolerance [25]. In this study, we use the neural network and logistic regression to investigate the relationship between three dynamic amino acid characteristics and the success rate for crystallization of 301 proteins from *A. thaliana* (Additional file 1: Table S1), and then compare the results with those obtained using each of 531 constant amino acid characteristics (Additional file 1: Table S2).

## Results and Discussion

The difference between constant amino acid characteristics documented in AAIndex [13] and dynamic characteristics [14–19] can be illustrated with two *A. thaliana* proteins [UniProtKB:P0C0B0, UniProtKB:Q8GW13]. Both proteins each contain 122 amino acids, but their amino acid compositions are different and their alignment reveals no similarity. Moreover, our knowledge of these two proteins is incomplete: P0C0B0 is an uncharacterized protein whereas Q8GW13 is a putative c-*myc* binding protein, which is suggestive of how these two proteins are represented using a constant amino acid characteristic from AAIndex [13] and a dynamic characteristic [14–19]. Table 1 presents this comparison of constant and dynamic characteristics. As can be seen, each protein had a different composition of amino acids (Table 1, columns 2 and 3). When arbitrarily using an amino acid characteristic, the CHAM830106 amino acid characteristic describes the number of bonds in the longest chain of amino acids [26]; for two proteins, the result is the same (Table 1, column 4). This was somewhat counterintuitive because each amino acid should have a different role in different proteins, at different positions, and with different neighboring amino acids. To modify this inflexibility, the characteristic was weighed by multiplying the number of corresponding amino acids (Table 1, columns 5 and 6). On the contrary, the two dynamic characteristics [14–19] varied and avoided inflexibility (Table 1, columns 7–10), which is an advantage over the constant amino acid characteristics documented in AAIndex [13].

**Table 1:** Comparison between constant and dynamic characteristics of amino acids

Amino acid	Number		CHAM830106^a^	CHAM830106 × number		Distribution probability^b^		Future composition (%)^c^	
	P0C0B0	Q8GW13		P0C0B0	Q8GW13	P0C0B0	Q8GW13	P0C0B0	Q8GW13
(Column 1)	(Column 2)	(Column 3)	(Column 4)	(Column 5)	(Column 6)	(Column 7)	(Column 8)	(Column 9)	(Column 10)
A	6	6	0	0	0	0.3472	0.2315	1.34	1.27
R	1	5	5	5	25	1.0000	0.2880	7.28	1.74
N	3	4	2	6	24	0.6667	0.5625	2.43	1.60
D	10	7	2	20	140	0.0127	0.2142	0.54	0.94
C	1	0	1	1	0	1.0000	0.0000	2.69	0.00
E	9	15	3	27	405	0.1967	0.0841	0.74	0.43
Q	2	6	3	6	36	0.5000	0.1543	2.24	0.99
G	7	4	0	0	0	0.2142	0.5625	0.99	1.56
H	4	5	3	12	60	0.1875	0.0640	0.76	0.92
I	9	2	2	18	36	0.1770	0.5000	0.86	2.70
L	6	16	2	12	192	0.3472	0.0568	1.79	0.64
K	17	14	4	68	952	0.0549	0.1178	0.29	0.40
M	3	2	3	9	18	0.6667	0.5000	0.90	1.02
F	10	2	4	40	80	0.1524	0.5000	0.37	1.79
P	4	3	0	0	0	0.5625	0.6667	1.17	2.02
S	8	13	1	8	104	0.2523	0.0515	0.99	0.62
T	8	6	1	8	48	0.2523	0.0386	1.03	1.18
W	1	0	5	5	0	1.0000	0.0000	0.60	0.00
Y	2	5	5	10	50	0.5000	0.2880	1.81	0.61
V	11	7	1	11	77	0.1077	0.1071	0.94	1.19

[UniProtKB:P0C0B0] and [UniProtKB:Q8GW13] are two *Arabidopsis thaliana* proteins

^a^CHAM830106 is an amino acid characteristic that describes the number of bonds in the longest chain of amino acids [26]

^b^The amino acid distribution probability is a dynamic characteristic computed according to the equation *r!/*(*q*_0_! × *q*_1_! × … × *q*_*n*_!) × *r!*/(*r*_1_! × *r*_2_! × … × *r*_*n*_!) × *n* ^*–r*^, where ! is the factorial, *r* is the number of a type of amino acid, *q* is the number of partitions with the same number of amino acids, and *n* is the number of partitions in the protein for a type of amino acid

^c^The future composition of amino acids in a protein was computed using the translation probability based on the relationship between RNA codons and their translated amino acids [14]

The previous studies which correlated the amino acid characteristics with the protein crystallization propensity [1–4] generally included all available amino acid characteristics together into a model. Certainly, such an approach dramatically enhanced the predictability of whether a protein was likely to be crystallized. However, the aim of this study was to determine the correlation between any dynamic characteristic [14–19] and crystallization propensity, and thus each individual characteristic of amino acids was used as a benchmark rather than all individual amino acid characteristics being used together in a model.

**Fig. 1:**
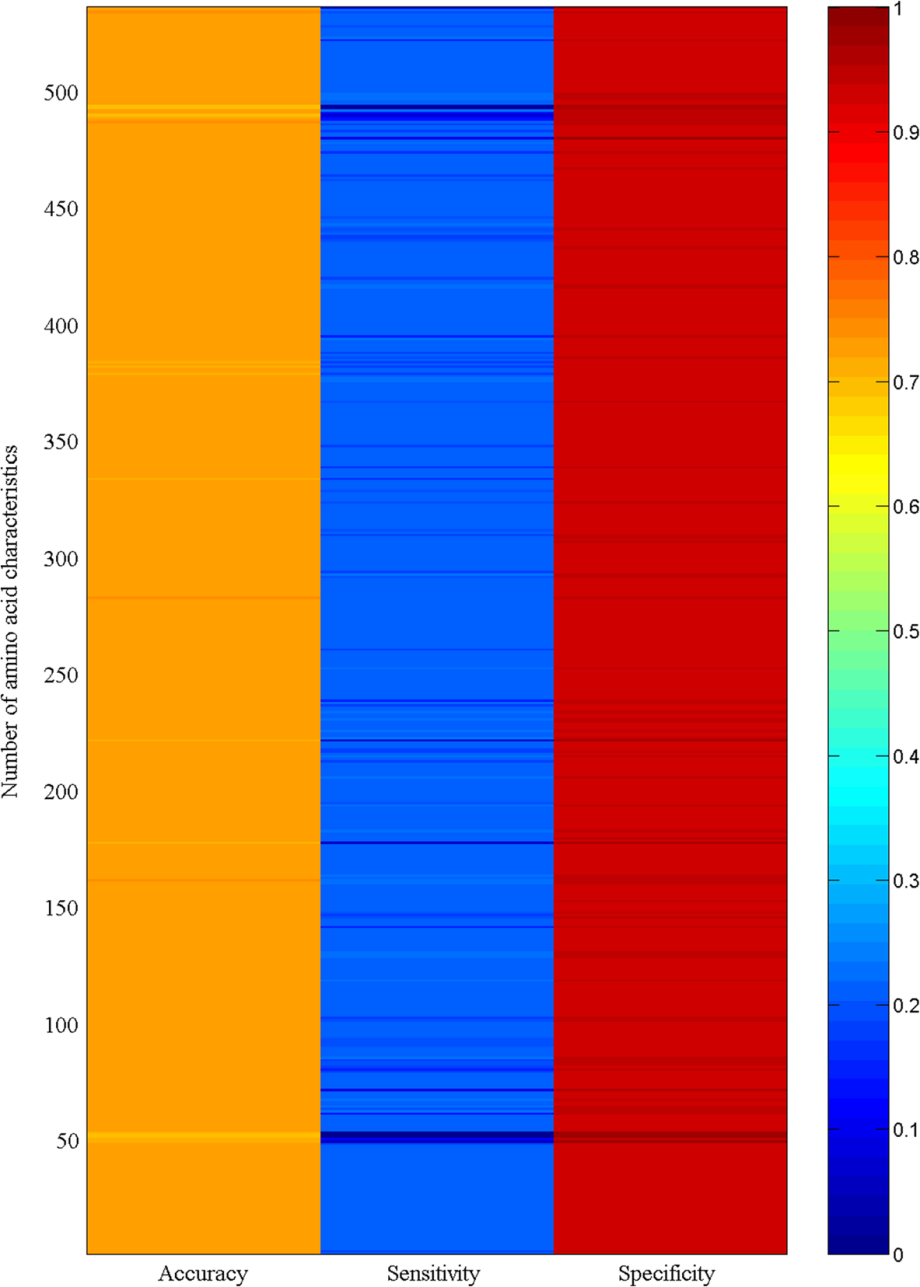
Heat map of the accuracy, sensitivity, and specificity of the crystallization propensity for 301 *A. thaliana* proteins predicted by logistic regression using each of 535 amino acid characteristics

**Fig. 2:**
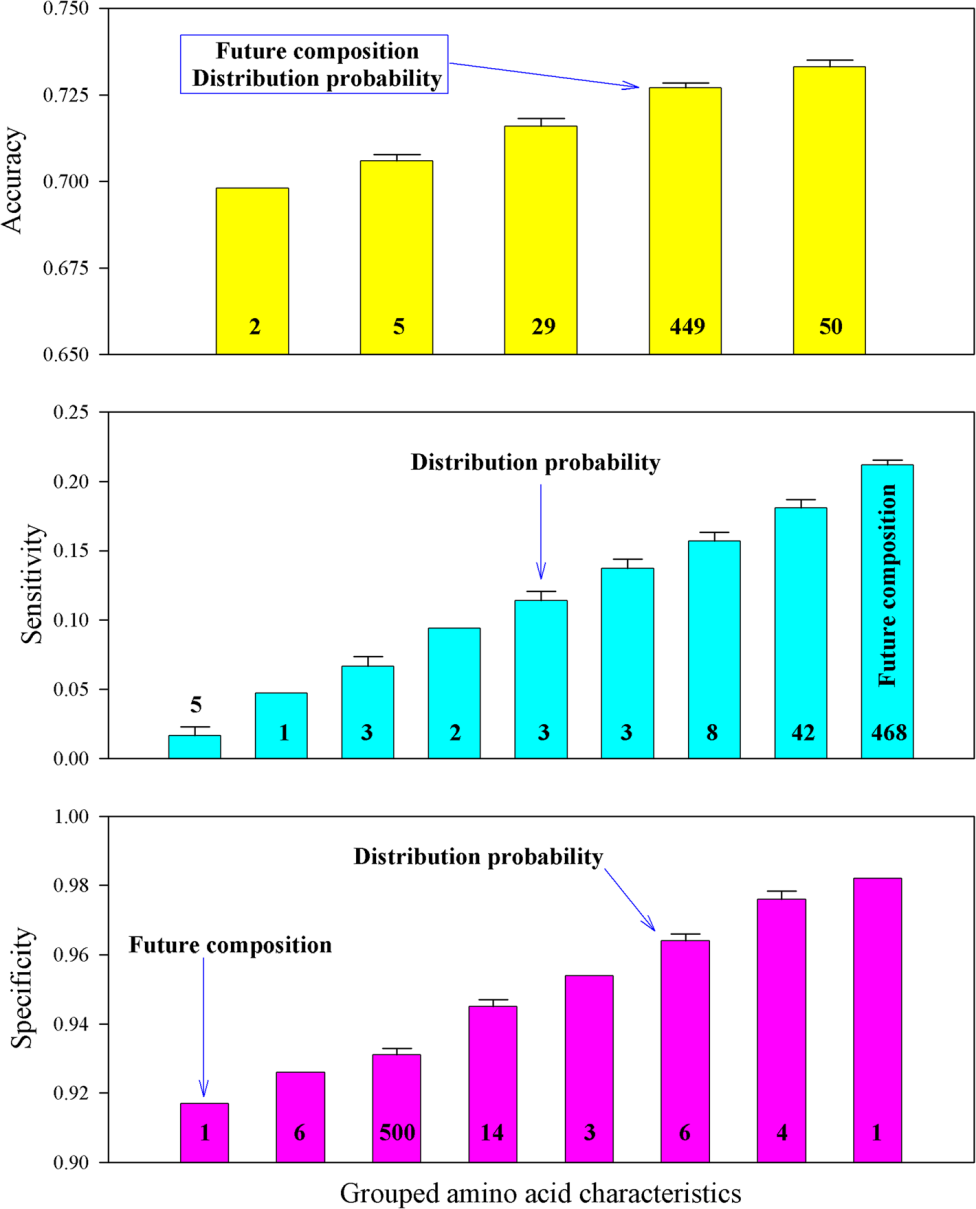
Comparison of the accuracy, sensitivity, and specificity of the crystallization propensity for 301 *A. thaliana* proteins predicted by logistic regression using 531 constant characteristics and two dynamic characteristics (future composition and distribution probability)

Figure 1 displays the heat map of the accuracy, sensitivity, and specificity of the crystallization propensity for 301 *A. thaliana* proteins predicted by logistic regression using each of 535 amino acid characteristics. It was obvious that different amino acid characteristics provided similar results with very low sensitivity and very high specificity. Figure 2 shows the comparison of the prediction results in Fig. 1. Each bar represents how many characteristics resulted in a similar accuracy, sensitivity, and specificity. For example, the first bar on the left-hand side in the upper panel indicates that two amino acid characteristics, FAUJ880109 and FAUJ880110, had the same accuracy of 0.698. Likewise, the second bar indicates that five amino acid characteristics had a similar accuracy (0.706 ± 0.002). Figure 2 demonstrates that many individual amino acid characteristics produced similar results, which is consistent with the study that showed the abundance of amino acid characteristics [27]. This figure also illustrates that two dynamic characteristics [14–19], future composition and distribution probability, were involved in protein crystallization.

**Fig. 3:**
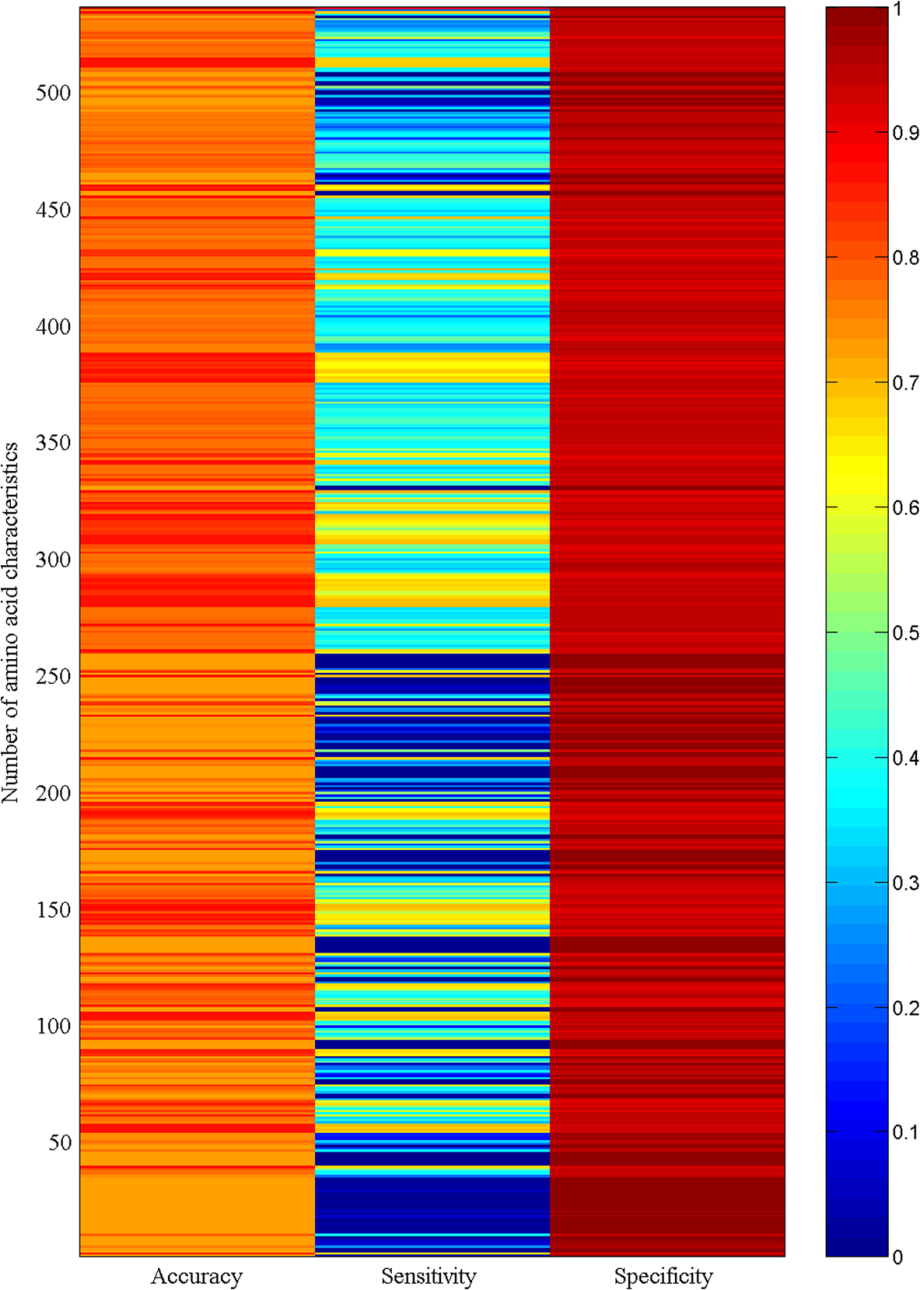
Heat map of the accuracy, sensitivity, and specificity of the crystallization propensity for 301 *A. thaliana* proteins fitted by neural network using each of 535 amino acid characteristics

**Fig. 4:**
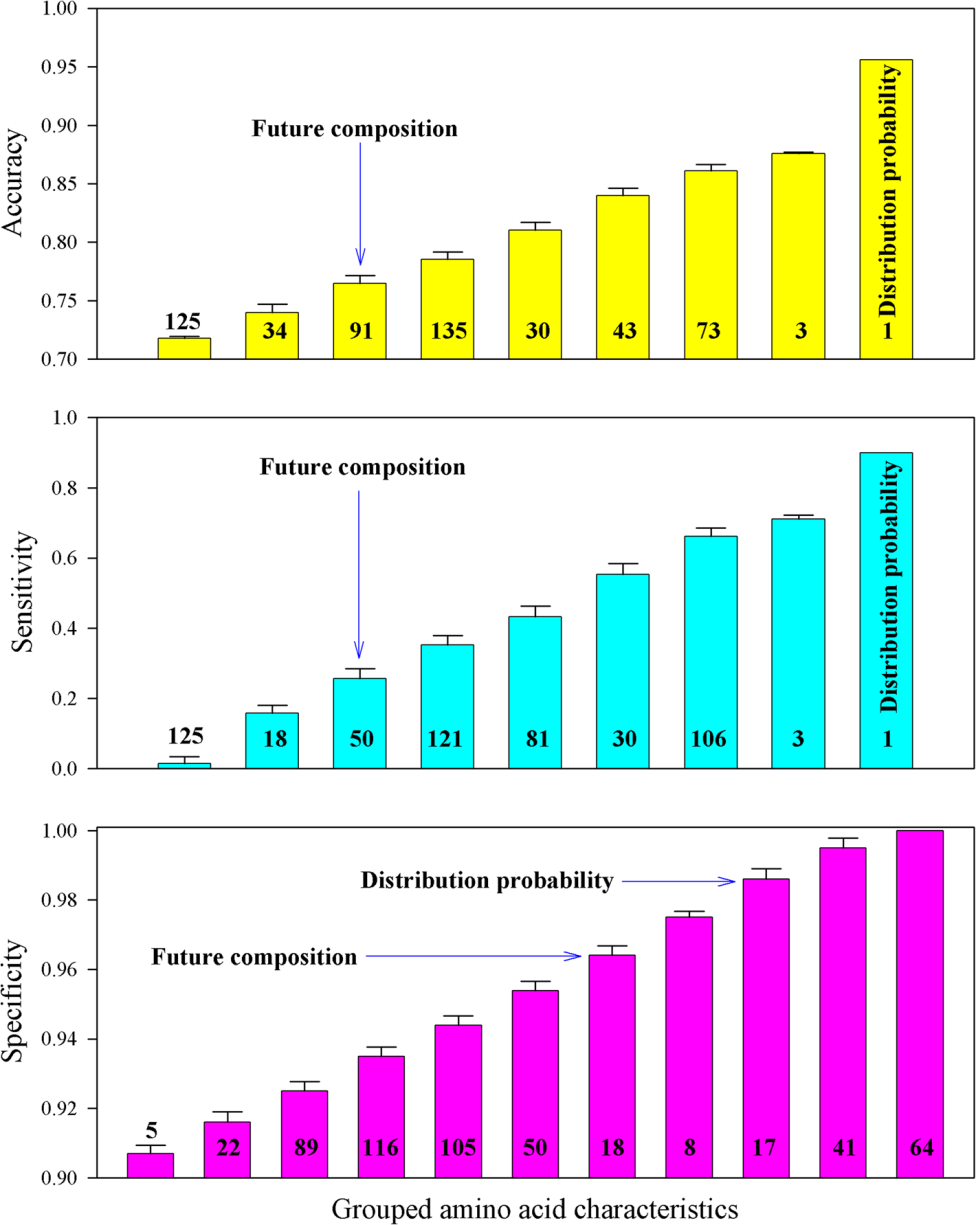
Comparison of the accuracy, sensitivity, and specificity of the crystallization propensity for 301 *A. thaliana* proteins fitted by neural network using 531 constant characteristics and two dynamic characteristics (future composition and distribution probability)

In the logistic regression model, the relationship appeared somewhat simple:

$$ P(y)=\frac{1}{1+{e}^{b_0+{b}_1{x}_1+\dots +{b}_{20}{x}_{20}}} $$

where *x*_1_, *x*_2_, … *x*_20_ are the characteristics for 20 types of amino acids, *y* is the crystallization success rate in 301 *A. thaliana* proteins (the crystallization success rate of a protein is either successful or unsuccessful so this value takes 1 for success and 0 for failure), and *b*_0_, *b*_1_, … *b*_20_ are logistic parameters. The neural network was therefore applied to model the relationship because, in principle, this model accounts for various implicit or explicit relationships [28, 29]. Figure 3 shows the heat map of the accuracy, sensitivity, and specificity of the crystallization propensity for 301 *A. thaliana* proteins fitted by the neural network using each of 535 amino acid characteristics. Clearly, the neural network could furthermore distinguish the difference among analyzed characteristics in relation to the crystallization propensity. Figure 4 shows the comparison of the fitting results in Fig. 3, which could be read as similar to those in Fig. 2. It is worth noting that the distribution probability gave the highest accuracy and sensitivity.

**Fig. 5:**
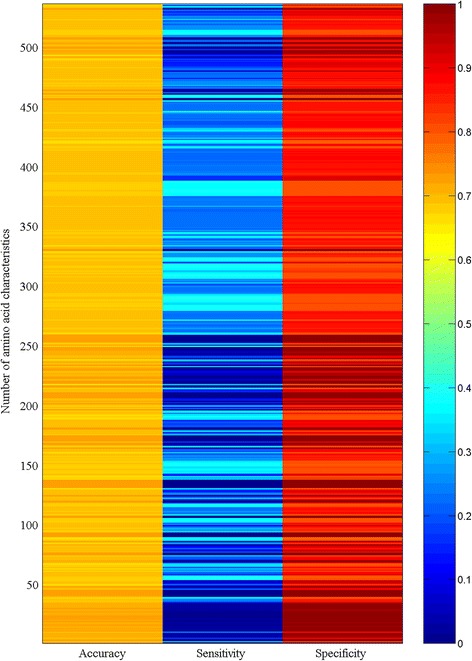
Heat map of the accuracy, sensitivity, and specificity of the crystallization propensity for 301 *A. thaliana* proteins validated by delete-1 jackknife validation using each of 535 amino acid characteristics

**Fig. 6:**
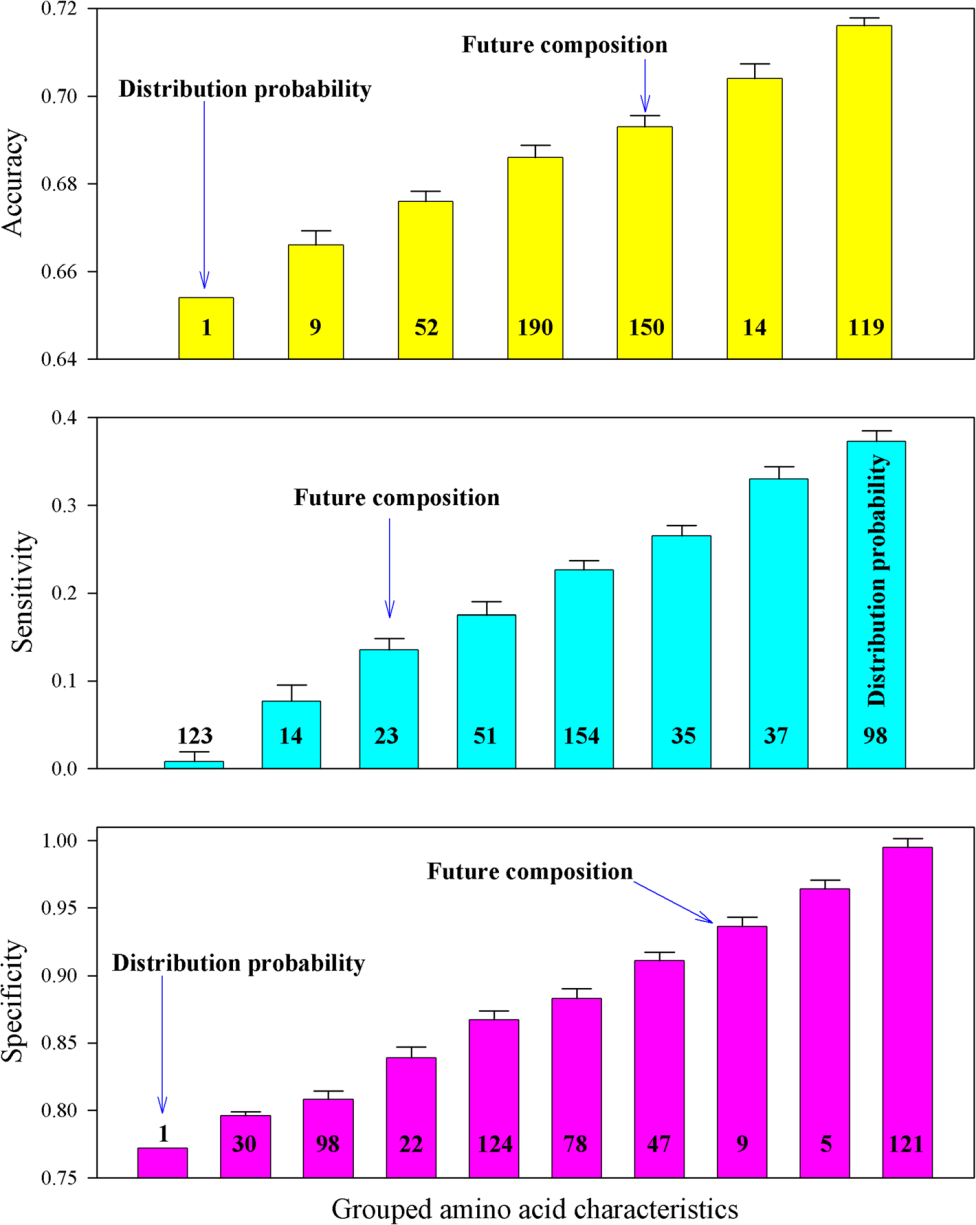
Comparison of the accuracy, sensitivity, and specificity of the crystallization propensity for 301 *A. thaliana* proteins validated by delete-1 jackknife validation using 531 constant characteristics and two dynamic characteristics (future composition and distribution probability)

Compared with the results obtained using the benchmark characteristics, Figs. 1, 2, 3, 4 suggest that dynamic characteristics of amino acids had a relationship with the crystallization propensity of the proteins from *A. thaliana*. Technically, the results in Figs. 1, 2, 3, 4 were obtained without dividing the database; that is, the model parameters obtained from all 301 *A. thaliana* proteins were used for predictions. This is generally the case in the first stage to determine whether a model is workable. Thereafter, the database should be divided into two groups, one for generating model parameters and the other for validation [30, 31]. Several methods of how to divide the dataset have been developed [31], one of which is jackknife validation [30–32]. The delete-1 jackknife validation in an *n*-sample dataset uses *n*—1 samples from the dataset to produce the model parameters and then makes a prediction for the deleted sample, so it requires *n* predictions rather than a few predictions as for other methods. This approach is considered better than that of other methods [31]. Figures 5 and 6 demonstrated the results of delete-1 jackknife validation obtained from a 10—1 neural network and their comparison. As can be seen, the predictions using dynamic characteristics were no worse than the predictions using the benchmark characteristics. A relationship between an amino acid characteristic and crystallization propensity can be judged with reference to their correlation coefficient. However, this is not sufficient for the modeling development, which asks whether this relationship is predictive or descriptive, and therefore the analysis of predictability is more meaningful [31]. Following this, the result implied that the dynamic characteristics [14–19] did have a certain relationship with crystallization propensity. A particular point from Fig. 6 is that the distribution probability did not appear superior because it was located in different areas. Because the specificities were identically high in the lower panel whereas the sensitivities were relatively low in the middle panel, the comparison should be directed to the sensitivity, where the distribution probability gave the best result.

**Fig. 7:**
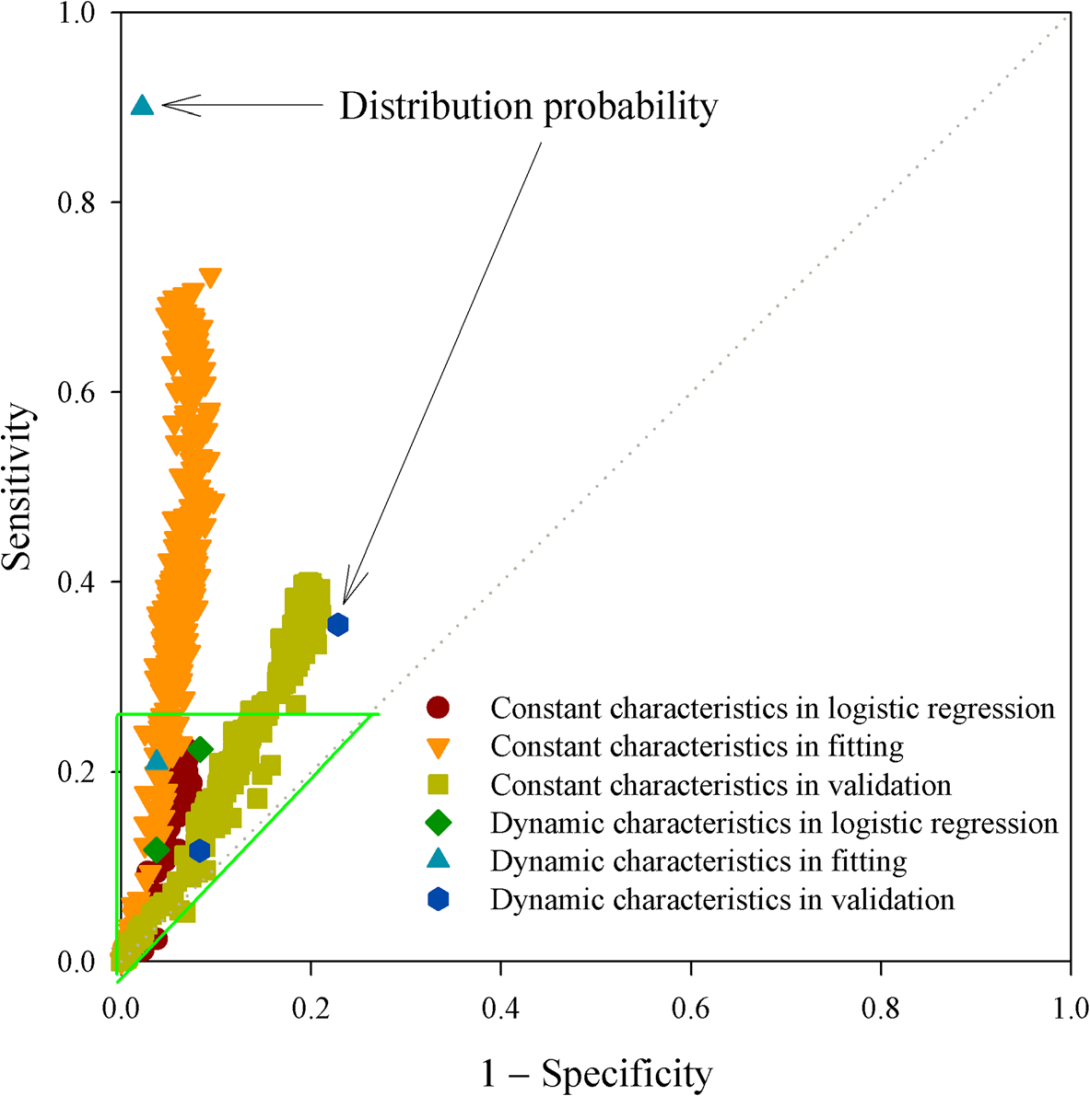
Comparison of sensitivity versus specificity by means of ROC analysis. *Diagonal line* is the line of indiscrimination indicating a completely random guess

To estimate a predictive model, comparison of the sensitivity versus the specificity can be assessed using receiver operating characteristic (ROC) curve analysis, which is mainly used for evaluation of various test methods [33, 34]. The prediction performance of amino acid characteristics can be further distinguished in Fig. 7. Although all predicted results were located in the upper-left triangle, indicating that their outcomes surpassed a random guess, some amino acid characteristics resulted in very low sensitivity and their results were located in the lower-left corner (triangular area). All of the results obtained from logistic regression scuttled inside this triangular area, indicating that logistic regression could not effectively screen the performance of different amino acid characteristics. The neural network model was more powerful for predicting the crystallization propensity of proteins. Compared with the benchmark, the dynamic characteristics of amino acids provided good prediction results for the crystallization propensity, and the distribution probability gave the best results in fitting and better results in validation.

**Fig. 8:**
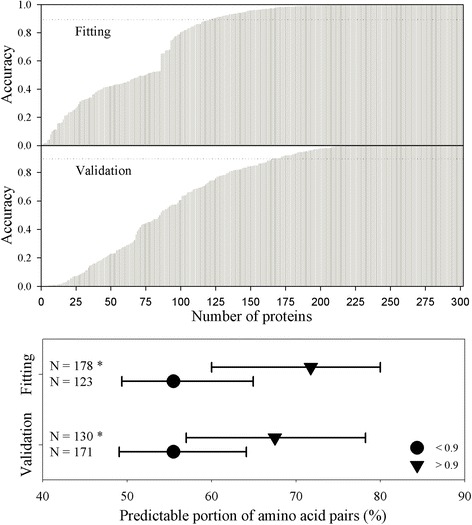
Crystallization accuracy of *A. thaliana* proteins obtained from model fitting (*upper panel*) and delete-1 jackknife validation (*middle panel*), and statistical comparison of their predictable portion of amino acid pairs (*lower panel*). *Dotted lines* indicate the cutoff point for separating the low accuracy from the high accuracy. Data presented as median with interquartile. *Statistically significant difference compared with the group of low accuracy at the *P* <0.001 level (Mann–Whitney rank sum test)

Only two dynamic characteristics [14–19], amino acid distribution probability and future composition, have so far been used. Another characteristic, the predictable portion of amino acid pairs, is mainly related to a whole protein. Figure 8 shows the results in this regard. In the two upper panels, each bar represents the accuracy obtained from fitting, where the predictions were conducted without dividing the database, and from delete-1 jackknife validation, where the prediction involved dividing the database. There were 301 bars in those two upper panels because this database contained 301 crystallized and noncrystallized proteins from *A. thaliana.* The 90 % accuracy was set as a cutoff point to be an acceptable accuracy. The predictive results were then ranked according to the predictable portion of *A. thaliana* portions, which brought about the statistical difference (Fig. 8, lower panel), suggesting that the larger the predictable portion, the better the prediction. Chen et al. [35] used the collocation of amino acid pairs to predict protein crystallization, and recently an ensemble method called SCMCRYS was developed to estimate the propensity scores of p-collocated amino acid pairs through a scoring card method [36], which provides the information that amino acid pairs do have some relationship with protein crystallization.

Many studies have explored new approaches to improve the prediction of protein crystallization propensity using various types of complemented features and complex ensemble classifiers. For example, AdaBoost uses two filter-mode feature selection methods to obtain 48 important features from 74 re-examined features [10]. PredPPCrys uses a comprehensive set of multifaceted sequence-derived features and combines a novel multistep feature selection strategy to predict the crystallization success [4]. RFCRYS used a random forest classifier [11]—including predicted surface ruggedness, hydrophobicity, side-chain entropy of surface residues and amino acid composition of the predicted protein surface—to improve the prediction of crystallization success [12]. Recently, support vector machines have been used to predict crystallization propensity of proteins based on sequence information [2, 4, 10, 36]. However, this study focused on determining whether dynamic characteristics of amino acids have some relation with protein crystallization, and thus a single characteristic should be used as the predictor rather than combined features. At this stage, simple classification models were suitable to conduct the performance, like the neural network. The reason why the dynamic characteristics worked better than constant amino acid characteristics in AAIndex [13] could be attributed to the fact that the dynamic characteristics take the amino acid spatial positions in a protein into account while other amino acid characteristics focus on the aspect of a single amino acid regardless of its position in a protein. On the contrary, the crystallization of a protein is more likely to be related to a protein structure in three-dimensional space rather than a certain aspect of a single amino acid.

## Conclusions

The results of this study were consistent with our previous studies [15–19] and confirmed that the dynamic characteristics [14–19] had a certain relationship with crystallization propensity of proteins. This appears reasonable because an amino acid should play different roles at different positions in a protein with different neighboring amino acids. However, constant characteristics of amino acids cannot reflect such changeable aspects. On the contrary, the dynamic characteristics of amino acids [14–19] do share changeable features, which should be more suitable to represent a protein. Dynamic characteristics could thus be useful to predict the propensity of protein crystallization.

## Methods

### Data

A total of 301 proteins from *A. thaliana* were found in TargetDB [37] under the purified criterion before 2011, 85 of which were also found under the crystallized criterion. These two criteria were once used to develop a web server for the prediction [8]. Detailed information for the 301 *A. thaliana* proteins is presented in Additional file 1: Table S1.

### Dynamic Characteristics for Both Protein and Amino Acids

The first dynamic characteristic is the amino acid distribution probability, which is based on the assumption that an amino acid’s position in a protein is analog to different colored balls in different holes, and corresponds to the problem of occupancy of subpopulations and partitions in probability [38], which computes the probability for each type of amino acids and is available online [39]. Two worked examples are presented in Table 1 (columns 7 and 8).

The second dynamic characteristic is the amino acid future composition. This characteristic is based on the relationship between RNA codons and their translated amino acids, suggesting the possibility that an amino acid may mutate into another amino acid (Additional file 1: Table S3) [40, 41], and therefore computes the future composition of a type of amino acid according to its current composition in a protein and mutating probability. Two worked examples are presented in Table 1 (columns 9 and 10). This characteristic can be calculated online [42].

The third dynamic characteristic is the amino acid pair predictability [14], which is based on the assumption that an amino acid involved in constructing an amino acid pair is independent of other amino acids and the probabilistic principle of multiplication should be applied. For example, a protein from *A. thaliana* [UniProtKB:P0C0B0] is composed of 122 amino acids, within which there are 17 lysines (K), seven glycines (G), and eight serines (S). Accordingly, the amino acid pair KK would appear twice in this protein (17/122 × 16/121 × 121 = 2.23). If we can find two KKs in this protein, they are predictable. The amino acid pair GS should not appear (7/122 × 8/121 × 121 = 0.46), but it appears three times in this protein so these amino acid pairs are unpredictable. In this manner, all amino acid pairs in a protein are classified either as predictable or as unpredictable. This protein has 75.25 % predictable and 24.75 % unpredictable amino acid pairs. Generally, the numbers of predictable and unpredictable pairs are different from protein to protein. This characteristic can be calculated online [43].

### Benchmark

The constant characteristics of amino acids are documented in AAIndex [13] and served as the benchmark to compare with the results obtained using dynamic characteristics. Currently, the AAIndex contains more than 540 characteristics to represent various aspects of the nature of amino acids, such as physicochemical characteristics, spatial characteristics [44], electronic characteristics [45], hydrophobic characteristics [46], and predictors for secondary structures [47]. There were 531 constant characteristics of amino acids used in this study and their detailed information is presented in Additional file 1: Table S2. The benchmark went through the same process as the dynamic characteristics: to code each amino acid in each *A. thaliana* protein with an amino acid characteristic from the AAIndex; to correlate each coded protein with its crystallization success rate using logistic regression and the neural network; to make predictions using the model parameters; and to compare the predictions based on an amino acid characteristic with the predictions based on a dynamic characteristic.

### Modeling

Both logistic regression and a 10—1 neural network were employed to model the relationship between an amino acid characteristic and success rate of protein crystallization. Because there were 20 types of amino acids, the relationship between 20 characteristics of amino acids (20 predictors) and the success rate of protein crystallization (one predicted function) was actually modeled.

### Statistical Analysis

The prediction of whether an *A. thaliana* protein could be crystallized was compared with what happened in realty. When an *A. thaliana* protein was predicted to be crystallized and was crystallized in reality, this prediction was classified as true positive (TP). When an *A. thaliana* protein was predicted to be not crystallized and was not crystallized in reality, this prediction was classified as true negative (TN). When an *A. thaliana* protein was predicted to be crystallized but was not crystallized in reality, this prediction was classified as false positive (FP). When an *A. thaliana* protein was predicted to be not crystallized but was crystallized in reality, this prediction was classified as false negative (FN). Thereafter, accuracy, sensitivity, and specificity can be computed as follows:

- Accuracy = (TP + TN) / (TP + FP + TN + FN) × 100
- Sensitivity = (TP) / (TP + FN) × 100
- Specificity = (TN) / (TN + FP) × 100

MatLab [29] was used to perform both logistic regression and the neural network. The ROC analysis was used to compare the sensitivity and specificity [48, 49]. Student’s *t* test was used for comparison, and *P* <0.05 was considered significant.

## Additional file

Additional file 1: Table S1. Presenting the 301 proteins from *A. Thaliana* used in this study. **Table S2.** Presenting the 535 characteristics of amino acids used in this study. **Table S3.** Presenting the amino acids and their translated amino acids. (DOC 780 kb)

## Abbreviations

FN: false negative
FP: false positive
ROC: receiver operating characteristic
TN: true negative
TP: true positive
