# Supplementary material for: Predicting Crystallization Propensity of Proteins from Arabidopsis Thaliana
Source: Biol Proced Online. 2015 Nov 23;17:16. doi: 10.1186/s12575-015-0029-3 (PMC4657326; doi:10.1186/s12575-015-0029-3)
Supplement: Additional file 1: Table S1. — Presenting the 301 proteins from A. Thaliana used in this study. Table S2. Presenting the 535 characteristics of amino acids used in this study. Table S3. Presenting the amino acids and their translated amino acids. (DOC 780 kb) [file 12575_2015_29_MOESM1_ESM.doc]

Table S1. List of 301 proteins from *Arabidopsis Thaliana* used in this study

| Order | ID in TargetDB | Laboratory | Length | State | Classification |
| --- | --- | --- | --- | --- | --- |
| 1 | atr001000161.1 | NON-RSGI | 51 | Purified | 0 |
| 2 | atr001002138.1 | NON-RSGI | 51 | Purified | 0 |
| 3 | atr001009308.1 | NON-RSGI | 51 | Purified | 0 |
| 4 | atr001109625.1 | NON-RSGI | 54 | Purified | 0 |
| 5 | atr001004282.1 | NON-RSGI | 58 | Purified | 0 |
| 6 | atr001000671.1 | NON-RSGI | 59 | Purified | 0 |
| 7 | atr001002820.1 | NON-RSGI | 60 | Purified | 0 |
| 8 | atr001010759.2 | NON-RSGI | 60 | Purified | 0 |
| 9 | atr001008714.1 | NON-RSGI | 61 | Purified | 0 |
| 10 | atr001006188.2 | NON-RSGI | 65 | Purified | 0 |
| 11 | atr001003181.1 | NON-RSGI | 67 | Purified | 0 |
| 12 | atr001010579.1 | NON-RSGI | 67 | Purified | 0 |
| 13 | GO.33909 | CESG | 67 | Purified | 0 |
| 14 | GO.7276 | CESG | 67 | Purified | 0 |
| 15 | atr001010103.1 | NON-RSGI | 71 | Purified | 0 |
| 16 | atr001010759.1 | NON-RSGI | 71 | Purified | 0 |
| 17 | GO.20081 | CESG | 71 | Purified | 0 |
| 18 | GO.9052 | CESG | 71 | Purified | 0 |
| 19 | AR3449A | NESG | 74 | Purified | 0 |
| 20 | AR3445A | NESG | 75 | Purified | 0 |
| 21 | atr001005336.1 | NON-RSGI | 76 | Purified | 0 |
| 22 | atr002000412.1 | NON-RSGI | 76 | Purified | 0 |
| 23 | GO.7474 | CESG | 78 | Purified | 0 |
| 24 | atr001008326.1 | NON-RSGI | 80 | Purified | 0 |
| 25 | atr001009297.1 | NON-RSGI | 80 | Purified | 0 |
| 26 | GO.33922 | CESG | 80 | Purified | 0 |
| 27 | atr002002959.1 | NON-RSGI | 81 | Purified | 0 |
| 28 | AR3433A | NESG | 84 | Purified | 0 |
| 29 | atr001006152.1 | NON-RSGI | 86 | Purified | 0 |
| 30 | AR3436A | NESG | 87 | Purified | 0 |
| 31 | GO.9098 | CESG | 87 | Purified | 0 |
| 32 | atr001006188.1 | NON-RSGI | 88 | Purified | 0 |
| 33 | GO.9161 | CESG | 89 | Purified | 0 |
| 34 | GO.11237 | CESG | 90 | Purified | 0 |
| 35 | GO.33930 | CESG | 92 | Purified | 0 |
| 36 | atr001004807.1 | NON-RSGI | 94 | Purified | 0 |
| 37 | GO.11308 | CESG | 95 | Purified | 0 |
| 38 | GO.33933 | CESG | 95 | Purified | 0 |
| 39 | atr001004705.1 | NON-RSGI | 98 | Purified | 0 |
| 40 | GO.6090 | CESG | 98 | Purified | 0 |
| 41 | atr001011428.1 | NON-RSGI | 100 | Purified | 0 |
| 42 | GO.6705 | CESG | 100 | Purified | 0 |
| 43 | atr001011678.1 | NON-RSGI | 101 | Purified | 0 |
| 44 | GO.33931 | CESG | 102 | Purified | 0 |
| 45 | GO.6042 | CESG | 103 | Crystallized | 1 |
| 46 | GO.7854 | CESG | 104 | Purified | 0 |
| 47 | atr001006273.1 | NON-RSGI | 105 | Purified | 0 |
| 48 | GO.78708 | CESG | 106 | Crystallized | 1 |
| 49 | GO.11962 | CESG | 107 | Purified | 0 |
| 50 | GO.13081 | CESG | 109 | Crystallized | 1 |
| 51 | GO.20961 | CESG | 110 | Purified | 0 |
| 52 | GO.22997 | CESG | 111 | Purified | 0 |
| 53 | GO.33932 | CESG | 112 | Purified | 0 |
| 54 | GO.14751 | CESG | 114 | Purified | 0 |
| 55 | atr001006482.1 | NON-RSGI | 117 | Purified | 0 |
| 56 | GO.15176 | CESG | 117 | Purified | 0 |
| 57 | GO.2953 | CESG | 117 | Purified | 0 |
| 58 | GO.12679 | CESG | 118 | Purified | 0 |
| 59 | GO.19021 | CESG | 118 | Purified | 0 |
| 60 | GO.20862 | CESG | 120 | Purified | 0 |
| 61 | atr001001566.1 | NON-RSGI | 121 | Purified | 0 |
| 62 | GO.5358 | CESG | 122 | Crystallized | 1 |
| 63 | GO.8772 | CESG | 122 | Purified | 0 |
| 64 | atr001008092.1 | NON-RSGI | 123 | Purified | 0 |
| 65 | atr001011378.1 | NON-RSGI | 124 | Purified | 0 |
| 66 | GO.111229 | CESG | 124 | Purified | 0 |
| 67 | atr001000337.1 | NON-RSGI | 127 | Purified | 0 |
| 68 | atr001003609.1 | NON-RSGI | 127 | Purified | 0 |
| 69 | GO.12174 | CESG | 127 | Purified | 0 |
| 70 | GO.8793 | CESG | 130 | Crystallized | 1 |
| 71 | GO.15028 | CESG | 131 | Crystallized | 1 |
| 72 | atr001010555.1 | NON-RSGI | 132 | Purified | 0 |
| 73 | GO.9984 | CESG | 132 | Purified | 0 |
| 74 | GO.5703 | CESG | 134 | Purified | 0 |
| 75 | GO.21134 | CESG | 135 | Purified | 0 |
| 76 | GO.24612 | CESG | 136 | Purified | 0 |
| 77 | GO.18555 | CESG | 139 | Purified | 0 |
| 78 | GO.78724 | CESG | 139 | Purified | 0 |
| 79 | GO.3781 | CESG | 140 | Crystallized | 1 |
| 80 | GO.33929 | CESG | 143 | Purified | 0 |
| 81 | GO.10862 | CESG | 144 | Purified | 0 |
| 82 | GO.13301 | CESG | 146 | Purified | 0 |
| 83 | GO.6108 | CESG | 146 | Purified | 0 |
| 84 | GO.13193 | CESG | 150 | Purified | 0 |
| 85 | GO.2361 | CESG | 151 | Crystallized | 1 |
| 86 | GO.9364 | CESG | 151 | Purified | 0 |
| 87 | GO.3977 | CESG | 152 | Purified | 0 |
| 88 | GO.14948 | CESG | 153 | Purified | 0 |
| 89 | atr001002493.1 | NON-RSGI | 155 | Purified | 0 |
| 90 | GO.11770 | CESG | 155 | Purified | 0 |
| 91 | GO.30556 | CESG | 156 | Purified | 0 |
| 92 | GO.13974 | CESG | 157 | Crystallized | 1 |
| 93 | GO.24256 | CESG | 158 | Crystallized | 1 |
| 94 | GO.3080 | CESG | 158 | Purified | 0 |
| 95 | GO.11624 | CESG | 161 | Purified | 0 |
| 96 | GO.6684 | CESG | 163 | Purified | 0 |
| 97 | GO.22446 | CESG | 165 | Purified | 0 |
| 98 | GO.22340 | CESG | 166 | Crystallized | 1 |
| 99 | GO.23028 | CESG | 166 | Crystallized | 1 |
| 100 | GO.6462 | CESG | 166 | Crystallized | 1 |
| 101 | GO.9943 | CESG | 166 | Purified | 0 |
| 102 | GO.10107 | CESG | 167 | Purified | 0 |
| 103 | GO.18354 | CESG | 167 | Crystallized | 1 |
| 104 | GO.12867 | CESG | 168 | Crystallized | 1 |
| 105 | GO.12401 | CESG | 169 | Purified | 0 |
| 106 | GO.3886 | CESG | 169 | Purified | 0 |
| 107 | GO.24315 | CESG | 170 | Purified | 0 |
| 108 | GO.11535 | CESG | 171 | Purified | 0 |
| 109 | GO.23603 | CESG | 171 | Crystallized | 1 |
| 110 | GO.6548 | CESG | 171 | Crystallized | 1 |
| 111 | GO.8490 | CESG | 171 | Purified | 0 |
| 112 | GO.12261 | CESG | 172 | Purified | 0 |
| 113 | GO.23605 | CESG | 172 | Crystallized | 1 |
| 114 | GO.12922 | CESG | 175 | Crystallized | 1 |
| 115 | GO.110778 | CESG | 176 | Crystallized | 1 |
| 116 | GO.3480 | CESG | 176 | Purified | 0 |
| 117 | GO.5649 | CESG | 177 | Purified | 0 |
| 118 | GO.16088 | CESG | 178 | Purified | 0 |
| 119 | GO.9733 | CESG | 178 | Crystallized | 1 |
| 120 | GO.13944 | CESG | 179 | Purified | 0 |
| 121 | GO.4577 | CESG | 179 | Purified | 0 |
| 122 | GO.12006 | CESG | 180 | Purified | 0 |
| 123 | GO.3010 | CESG | 180 | Purified | 0 |
| 124 | GO.16072 | CESG | 183 | Purified | 0 |
| 125 | GO.23366 | CESG | 183 | Crystallized | 1 |
| 126 | GO.110777 | CESG | 184 | Crystallized | 1 |
| 127 | GO.22965 | CESG | 185 | Purified | 0 |
| 128 | GO.22294 | CESG | 186 | Crystallized | 1 |
| 129 | GO.1646 | CESG | 189 | Crystallized | 1 |
| 130 | GO.14298 | CESG | 191 | Purified | 0 |
| 131 | GO.30687 | CESG | 191 | Purified | 0 |
| 132 | GO.2041 | CESG | 192 | Purified | 0 |
| 133 | GO.695 | CESG | 192 | Crystallized | 1 |
| 134 | GO.690 | CESG | 194 | Purified | 0 |
| 135 | GO.14480 | CESG | 198 | Purified | 0 |
| 136 | GO.7274 | CESG | 198 | Purified | 0 |
| 137 | GO.6058 | CESG | 202 | Purified | 0 |
| 138 | GO.14481 | CESG | 205 | Purified | 0 |
| 139 | GO.22116 | CESG | 206 | Crystallized | 1 |
| 140 | GO.14546 | CESG | 208 | Purified | 0 |
| 141 | GO.21151 | CESG | 208 | Purified | 0 |
| 142 | NYSGXRC-11003o | NYSGXRC | 208 | Crystallized | 1 |
| 143 | GO.11100 | CESG | 209 | Crystallized | 1 |
| 144 | GO.12688 | CESG | 209 | Crystallized | 1 |
| 145 | GO.34352 | CESG | 210 | Purified | 0 |
| 146 | GO.34354 | CESG | 210 | Purified | 0 |
| 147 | GO.4031 | CESG | 212 | Purified | 0 |
| 148 | GO.19912 | CESG | 215 | Crystallized | 1 |
| 149 | GO.3145 | CESG | 215 | Crystallized | 1 |
| 150 | GO.605 | CESG | 215 | Crystallized | 1 |
| 151 | GO.34355 | CESG | 216 | Crystallized | 1 |
| 152 | GO.34356 | CESG | 216 | Crystallized | 1 |
| 153 | GO.5555 | CESG | 216 | Purified | 0 |
| 154 | GO.24556 | CESG | 217 | Crystallized | 1 |
| 155 | GO.12240 | CESG | 221 | Purified | 0 |
| 156 | GO.4553 | CESG | 221 | Purified | 0 |
| 157 | GO.22148 | CESG | 224 | Purified | 0 |
| 158 | GO.6728 | CESG | 225 | Purified | 0 |
| 159 | GO.12338 | CESG | 226 | Crystallized | 1 |
| 160 | GO.2118 | CESG | 227 | Purified | 0 |
| 161 | GO.2638 | CESG | 228 | Purified | 0 |
| 162 | GO.12325 | CESG | 230 | Crystallized | 1 |
| 163 | GO.17129 | CESG | 235 | Purified | 0 |
| 164 | GO.23752 | CESG | 235 | Purified | 0 |
| 165 | GO.11619 | CESG | 236 | Purified | 0 |
| 166 | GO.2521 | CESG | 237 | Purified | 0 |
| 167 | GO.14895 | CESG | 239 | Purified | 0 |
| 168 | GO.10054 | CESG | 241 | Crystallized | 1 |
| 169 | GO.17262 | CESG | 244 | Purified | 0 |
| 170 | GO.1769 | CESG | 244 | Purified | 0 |
| 171 | GO.3437 | CESG | 245 | Purified | 0 |
| 172 | GO.17035 | CESG | 246 | Purified | 0 |
| 173 | GO.8055 | CESG | 246 | Crystallized | 1 |
| 174 | GO.11015 | CESG | 247 | Purified | 0 |
| 175 | GO.2059 | CESG | 248 | Purified | 0 |
| 176 | GO.22814 | CESG | 248 | Purified | 0 |
| 177 | GO.23901 | CESG | 248 | Purified | 0 |
| 178 | GO.17393 | CESG | 251 | Purified | 0 |
| 179 | GO.15839 | CESG | 253 | Crystallized | 1 |
| 180 | GO.23662 | CESG | 253 | Purified | 0 |
| 181 | APC041 | MCSG | 254 | Purified | 0 |
| 182 | GO.15838 | CESG | 254 | Crystallized | 1 |
| 183 | GO.1652 | CESG | 254 | Crystallized | 1 |
| 184 | GO.1764 | CESG | 256 | Crystallized | 1 |
| 185 | GO.4980 | CESG | 256 | Crystallized | 1 |
| 186 | GO.91563 | CESG | 258 | Purified | 0 |
| 187 | AR2241 | NESG | 260 | Crystallized | 1 |
| 188 | GO.22797 | CESG | 260 | Crystallized | 1 |
| 189 | GO.6199 | CESG | 260 | Purified | 0 |
| 190 | GO.21372 | CESG | 261 | Purified | 0 |
| 191 | GO.19168 | CESG | 262 | Purified | 0 |
| 192 | GO.10406 | CESG | 263 | Purified | 0 |
| 193 | GO.3577 | CESG | 263 | Crystallized | 1 |
| 194 | GO.6917 | CESG | 263 | Crystallized | 1 |
| 195 | GO.22716 | CESG | 264 | Purified | 0 |
| 196 | GO.11131 | CESG | 265 | Crystallized | 1 |
| 197 | GO.10170 | CESG | 266 | Purified | 0 |
| 198 | GO.7490 | CESG | 270 | Purified | 0 |
| 199 | GO.19924 | CESG | 272 | Purified | 0 |
| 200 | GO.5436 | CESG | 276 | Purified | 0 |
| 201 | GO.12200 | CESG | 281 | Crystallized | 1 |
| 202 | GO.23723 | CESG | 285 | Purified | 0 |
| 203 | GO.23975 | CESG | 286 | Crystallized | 1 |
| 204 | GO.7556 | CESG | 286 | Purified | 0 |
| 205 | GO.15517 | CESG | 287 | Purified | 0 |
| 206 | GO.6078 | CESG | 287 | Purified | 0 |
| 207 | GO.21130 | CESG | 291 | Purified | 0 |
| 208 | GO.16226 | CESG | 294 | Purified | 0 |
| 209 | GO.29869 | CESG | 294 | Purified | 0 |
| 210 | GO.34361 | CESG | 295 | Purified | 0 |
| 211 | GO.34362 | CESG | 295 | Purified | 0 |
| 212 | GO.1547 | CESG | 296 | Purified | 0 |
| 213 | GO.12798 | CESG | 300 | Purified | 0 |
| 214 | NYSGXRC-10499a | NYSGXRC | 300 | Purified | 0 |
| 215 | GO.10721 | CESG | 305 | Purified | 0 |
| 216 | GO.18476 | CESG | 308 | Purified | 0 |
| 217 | atr002004172.1 | NON-RSGI | 309 | Crystallized | 1 |
| 218 | GO.20981 | CESG | 312 | Purified | 0 |
| 219 | GO.17357 | CESG | 315 | Purified | 0 |
| 220 | GO.6763 | CESG | 316 | Purified | 0 |
| 221 | GO.11773 | CESG | 317 | Purified | 0 |
| 222 | GO.11775 | CESG | 317 | Purified | 0 |
| 223 | GO.1255 | CESG | 317 | Purified | 0 |
| 224 | GO.33429 | CESG | 317 | Purified | 0 |
| 225 | GO.4020 | CESG | 317 | Crystallized | 1 |
| 226 | GO.16343 | CESG | 318 | Purified | 0 |
| 227 | GO.8893 | CESG | 318 | Purified | 0 |
| 228 | GO.7938 | CESG | 320 | Purified | 0 |
| 229 | GO.81882 | CESG | 321 | Purified | 0 |
| 230 | GO.10931 | CESG | 322 | Purified | 0 |
| 231 | GO.21674 | CESG | 324 | Purified | 0 |
| 232 | GO.33237 | CESG | 324 | Purified | 0 |
| 233 | GO.9639 | CESG | 324 | Crystallized | 1 |
| 234 | GO.8254 | CESG | 325 | Crystallized | 1 |
| 235 | NYSGXRC-10016c | NYSGXRC | 325 | Crystallized | 1 |
| 236 | GO.29367 | CESG | 326 | Purified | 0 |
| 237 | GO.31655 | CESG | 326 | Purified | 0 |
| 238 | GO.3451 | CESG | 326 | Purified | 0 |
| 239 | GO.7312 | CESG | 326 | Crystallized | 1 |
| 240 | GO.21541 | CESG | 329 | Purified | 0 |
| 241 | GO.13167 | CESG | 330 | Crystallized | 1 |
| 242 | GO.14486 | CESG | 331 | Purified | 0 |
| 243 | GO.20607 | CESG | 332 | Purified | 0 |
| 244 | GO.14640 | CESG | 333 | Purified | 0 |
| 245 | GO.4295 | CESG | 333 | Crystallized | 1 |
| 246 | GO.18218 | CESG | 334 | Purified | 0 |
| 247 | GO.18651 | CESG | 334 | Crystallized | 1 |
| 248 | GO.3851 | CESG | 334 | Crystallized | 1 |
| 249 | GO.102288 | CESG | 335 | Purified | 0 |
| 250 | GO.6759 | CESG | 335 | Purified | 0 |
| 251 | GO.6760 | CESG | 335 | Purified | 0 |
| 252 | GO.24901 | CESG | 336 | Purified | 0 |
| 253 | GO.1419 | CESG | 340 | Purified | 0 |
| 254 | GO.24957 | CESG | 340 | Purified | 0 |
| 255 | GO.22013 | CESG | 346 | Purified | 0 |
| 256 | GO.23034 | CESG | 348 | Crystallized | 1 |
| 257 | GO.2840 | CESG | 350 | Purified | 0 |
| 258 | GO.23169 | CESG | 351 | Crystallized | 1 |
| 259 | GO.14940 | CESG | 354 | Crystallized | 1 |
| 260 | GO.23423 | CESG | 356 | Crystallized | 1 |
| 261 | GO.8195 | CESG | 357 | Crystallized | 1 |
| 262 | GO.6283 | CESG | 358 | Purified | 0 |
| 263 | GO.9383 | CESG | 359 | Crystallized | 1 |
| 264 | GO.19812 | CESG | 362 | Crystallized | 1 |
| 265 | GO.17740 | CESG | 363 | Purified | 0 |
| 266 | GO.110779 | CESG | 364 | Crystallized | 1 |
| 267 | GO.3073 | CESG | 372 | Crystallized | 1 |
| 268 | GO.22301 | CESG | 374 | Crystallized | 1 |
| 269 | GO.3074 | CESG | 374 | Crystallized | 1 |
| 270 | GO.8435 | CESG | 374 | Crystallized | 1 |
| 271 | GO.24674 | CESG | 383 | Crystallized | 1 |
| 272 | GO.12079 | CESG | 387 | Purified | 0 |
| 273 | GO.8800 | CESG | 387 | Purified | 0 |
| 274 | GO.11932 | CESG | 389 | Purified | 0 |
| 275 | GO.8210 | CESG | 391 | Crystallized | 1 |
| 276 | AR3710 | NESG | 398 | Purified | 0 |
| 277 | GO.23321 | CESG | 407 | Purified | 0 |
| 278 | APC41718.0 | MCSG | 415 | Purified | 0 |
| 279 | GO.92 | CESG | 420 | Crystallized | 1 |
| 280 | GO.17621 | CESG | 434 | Crystallized | 1 |
| 281 | GO.5187 | CESG | 435 | Crystallized | 1 |
| 282 | GO.22206 | CESG | 438 | Crystallized | 1 |
| 283 | GO.22207 | CESG | 439 | Crystallized | 1 |
| 284 | NYSGXRC-10413l | NYSGXRC | 440 | Purified | 0 |
| 285 | atr001004097.1 | NON-RSGI | 441 | Purified | 0 |
| 286 | GO.34358 | CESG | 451 | Purified | 0 |
| 287 | GO.24707 | CESG | 454 | Purified | 0 |
| 288 | GO.16373 | CESG | 461 | Purified | 0 |
| 289 | GO.20938 | CESG | 461 | Purified | 0 |
| 290 | GO.14914 | CESG | 469 | Crystallized | 1 |
| 291 | GO.22477 | CESG | 470 | Purified | 0 |
| 292 | GO.23749 | CESG | 491 | Purified | 0 |
| 293 | GO.20879 | CESG | 523 | Purified | 0 |
| 294 | GO.29544 | CESG | 524 | Crystallized | 1 |
| 295 | NYSGXRC-11039a | NYSGXRC | 648 | Purified | 0 |
| 296 | NYSGXRC-11047a | NYSGXRC | 674 | Purified | 0 |
| 297 | NYSGXRC-T750 | NYSGXRC | 687 | Crystallized | 1 |
| 298 | GO.1726 | CESG | 697 | Purified | 0 |
| 299 | atr001007933.1 | NON-RSGI | 712 | Purified | 0 |
| 300 | GO.13591 | CESG | 824 | Purified | 0 |
| 301 | GO.11648 | CESG | 839 | Crystallized | 1 |

Table S2. List of 535 characteristics of amino acids used in this study

| Order | Accession number | Class I | Description | Author(s) |
| --- | --- | --- | --- | --- |
| 1 | DAYM780101 | Composition | Amino acid composition | Dayhoff et al., 1978a |
| 2 | GRAR740101 | Composition | Composition | Grantham, 1974 |
| 3 | JUNJ780101 | Composition | Sequence frequency | Jungck, 1978 |
| 4 | NAKH900101 | Composition | AA composition of total proteins | Nakashima et al., 1990 |
| 5 | NAKH900102 | Composition | SD of AA composition of total proteins | Nakashima et al., 1990 |
| 6 | NAKH900103 | Composition | AA composition of mt-proteins | Nakashima et al., 1990 |
| 7 | NAKH900105 | Composition | AA composition of mt-proteins from animal | Nakashima et al., 1990 |
| 8 | NAKH900107 | Composition | AA composition of mt-proteins from fungi and plant | Nakashima et al., 1990 |
| 9 | NAKH900109 | Composition | AA composition of membrane proteins | Nakashima et al., 1990 |
| 10 | NAKH900113 | Composition | Ratio of average and computed composition | Nakashima et al., 1990 |
| 11 | NAKH920101 | Composition | AA composition of CYT of single-spanning proteins | Nakashima-Nishikawa, 1992 |
| 12 | NAKH920102 | Composition | AA composition of CYT2 of single-spanning proteins | Nakashima-Nishikawa, 1992 |
| 13 | NAKH920103 | Composition | AA composition of EXT of single-spanning proteins | Nakashima-Nishikawa, 1992 |
| 14 | NAKH920104 | Composition | AA composition of EXT2 of single-spanning proteins | Nakashima-Nishikawa, 1992 |
| 15 | NAKH920105 | Composition | AA composition of MEM of single-spanning proteins | Nakashima-Nishikawa, 1992 |
| 16 | NAKH920106 | Composition | AA composition of CYT of multi-spanning proteins | Nakashima-Nishikawa, 1992 |
| 17 | NAKH920107 | Composition | AA composition of EXT of multi-spanning proteins | Nakashima-Nishikawa, 1992 |
| 18 | NAKH920108 | Composition | AA composition of MEM of multi-spanning proteins | Nakashima-Nishikawa, 1992 |
| 19 | CEDJ970101 | Composition | Composition of amino acids in extracellular proteins (percent) | Cedano et al., 1997 |
| 20 | CEDJ970102 | Composition | Composition of amino acids in anchored proteins (percent) | Cedano et al., 1997 |
| 21 | CEDJ970103 | Composition | Composition of amino acids in membrane proteins (percent) | Cedano et al., 1997 |
| 22 | CEDJ970104 | Composition | Composition of amino acids in intracellular proteins (percent) | Cedano et al., 1997 |
| 23 | CEDJ970105 | Composition | Composition of amino acids in nuclear proteins (percent) | Cedano et al., 1997 |
| 24 | FUKS010101 | Composition | Surface composition of amino acids in intracellular proteins of thermophiles (percent) | Fukuchi-Nishikawa, 2001 |
| 25 | FUKS010102 | Composition | Surface composition of amino acids in intracellular proteins of mesophiles (percent) | Fukuchi-Nishikawa, 2001 |
| 26 | FUKS010103 | Composition | Surface composition of amino acids in extracellular proteins of mesophiles (percent) | Fukuchi-Nishikawa, 2001 |
| 27 | FUKS010104 | Composition | Surface composition of amino acids in nuclear proteins (percent) | Fukuchi-Nishikawa, 2001 |
| 28 | FUKS010105 | Composition | Interior composition of amino acids in intracellular proteins of thermophiles (percent) | Fukuchi-Nishikawa, 2001 |
| 29 | FUKS010106 | Composition | Interior composition of amino acids in intracellular proteins of mesophiles (percent) | Fukuchi-Nishikawa, 2001 |
| 30 | FUKS010107 | Composition | Interior composition of amino acids in extracellular proteins of mesophiles (percent) | Fukuchi-Nishikawa, 2001 |
| 31 | FUKS010108 | Composition | Interior composition of amino acids in nuclear proteins (percent) | Fukuchi-Nishikawa, 2001 |
| 32 | FUKS010109 | Composition | Entire chain composition of amino acids in intracellular proteins of thermophiles (percent) | Fukuchi-Nishikawa, 2001 |
| 33 | FUKS010110 | Composition | Entire chain composition of amino acids in intracellular proteins of mesophiles (percent) | Fukuchi-Nishikawa, 2001 |
| 34 | FUKS010111 | Composition | Entire chain composition of amino acids in extracellular proteins of mesophiles (percent) | Fukuchi-Nishikawa, 2001 |
| 35 | JUKT750101 | Composition | Amino acid distribution | Jukes et al., 1975 |
| 36 | NAKH900104 | Composition | Normalized composition of mt-proteins | Nakashima et al., 1990 |
| 37 | NAKH900106 | Composition | Normalized composition from animal | Nakashima et al., 1990 |
| 38 | NAKH900108 | Composition | Normalized composition from fungi and plant | Nakashima et al., 1990 |
| 39 | NAKH900110 | Composition | Normalized composition of membrane proteins | Nakashima et al., 1990 |
| 40 | FUKS010112 | Composition | Entire chain composition of amino acids in nuclear proteins (percent) | Fukuchi-Nishikawa, 2001 |
| 41 | BIOV880101 | Physicochemical property | Information value for accessibility; average fraction 35% | Biou et al., 1988 |
| 42 | BIOV880102 | Physicochemical property | Information value for accessibility; average fraction 23% | Biou et al., 1988 |
| 43 | CHOC760101 | Physicochemical property | Residue accessible surface area in tripeptide | Chothia, 1976 |
| 44 | CHOC760102 | Physicochemical property | Residue accessible surface area in folded protein | Chothia, 1976 |
| 45 | JANJ780101 | Physicochemical property | Average accessible surface area | Janin et al., 1978 |
| 46 | JANJ790101 | Physicochemical property | Ratio of buried and accessible molar fractions | Janin, 1979 |
| 47 | PONP800107 | Physicochemical property | Accessibility reduction ratio | Ponnuswamy et al., 1980 |
| 48 | RADA880106 | Physicochemical property | Accessible surface area | Radzicka-Wolfenden, 1988 |
| 49 | CHAM830107 | Physicochemical property | A parameter of charge transfer capability | Charton-Charton, 1983 |
| 50 | CHAM830108 | Physicochemical property | A parameter of charge transfer donor capability | Charton-Charton, 1983 |
| 51 | FAUJ880111 | Physicochemical property | Positive charge | Fauchere et al., 1988 |
| 52 | FAUJ880112 | Physicochemical property | Negative charge | Fauchere et al., 1988 |
| 53 | KLEP840101 | Physicochemical property | Net charge | Klein et al., 1984 |
| 54 | SNEP660101 | Physicochemical property | Principal component I | Sneath, 1966 |
| 55 | SNEP660102 | Physicochemical property | Principal component II | Sneath, 1966 |
| 56 | SNEP660103 | Physicochemical property | Principal component III | Sneath, 1966 |
| 57 | SNEP660104 | Physicochemical property | Principal component IV | Sneath, 1966 |
| 58 | WOLS870101 | Physicochemical property | Principal property value z1 | Wold et al., 1987 |
| 59 | WOLS870102 | Physicochemical property | Principal property value z2 | Wold et al., 1987 |
| 60 | WOLS870103 | Physicochemical property | Principal property value z3 | Wold et al., 1987 |
| 61 | FAUJ880108 | Physicochemical property | Localized electrical effect | Fauchere et al., 1988 |
| 62 | BULH740101 | Physicochemical property | Transfer free energy to surface | Bull-Breese, 1974 |
| 63 | CHAM820102 | Physicochemical property | Free energy of solution in water, kcal/mole | Charton-Charton, 1982 |
| 64 | EISD860101 | Physicochemical property | Solvation free energy | Eisenberg-McLachlan, 1986 |
| 65 | GUYH850101 | Physicochemical property | Partition energy | Guy, 1985 |
| 66 | JANJ790102 | Physicochemical property | Transfer free energy | Janin, 1979 |
| 67 | LAWE840101 | Physicochemical property | Transfer free energy, CHP/water | Lawson et al., 1984 |
| 68 | MIYS850101 | Physicochemical property | Effective partition energy | Miyazawa-Jernigan, 1985 |
| 69 | NAKH900111 | Physicochemical property | Transmembrane regions of non-mt-proteins | Nakashima et al., 1990 |
| 70 | NAKH900112 | Physicochemical property | Transmembrane regions of mt-proteins | Nakashima et al., 1990 |
| 71 | NOZY710101 | Physicochemical property | Transfer energy, organic solvent/water | Nozaki-Tanford, 1971 |
| 72 | OOBM770101 | Physicochemical property | Average non-bonded energy per atom | Oobatake-Ooi, 1977 |
| 73 | OOBM770102 | Physicochemical property | Short and medium range non-bonded energy per atom | Oobatake-Ooi, 1977 |
| 74 | OOBM770103 | Physicochemical property | Long range non-bonded energy per atom | Oobatake-Ooi, 1977 |
| 75 | OOBM770104 | Physicochemical property | Average non-bonded energy per residue | Oobatake-Ooi, 1977 |
| 76 | OOBM770105 | Physicochemical property | Short and medium range non-bonded energy per residue | Oobatake-Ooi, 1977 |
| 77 | OOBM850103 | Physicochemical property | Optimized transfer energy parameter | Oobatake et al., 1985 |
| 78 | OOBM850104 | Physicochemical property | Optimized average non-bonded energy per atom | Oobatake et al., 1985 |
| 79 | RADA880101 | Physicochemical property | Transfer free energy from chx to wat | Radzicka-Wolfenden, 1988 |
| 80 | RADA880102 | Physicochemical property | Transfer free energy from oct to wat | Radzicka-Wolfenden, 1988 |
| 81 | RADA880103 | Physicochemical property | Transfer free energy from vap to chx | Radzicka-Wolfenden, 1988 |
| 82 | RADA880104 | Physicochemical property | Transfer free energy from chx to oct | Radzicka-Wolfenden, 1988 |
| 83 | RADA880105 | Physicochemical property | Transfer free energy from vap to oct | Radzicka-Wolfenden, 1988 |
| 84 | RADA880107 | Physicochemical property | Energy transfer from out to in (95 % buried) | Radzicka-Wolfenden, 1988 |
| 85 | SIMZ760101 | Physicochemical property | Transfer free energy | Simon, 1976, cited by Charton-Charton , 1982 |
| 86 | VHEG790101 | Physicochemical property | Transfer free energy to lipophilic phase | von Heijne-Blomberg, 1979 |
| 87 | WERD780102 | Physicochemical property | Free energy change of epsilon(i) to epsilon(ex) | Wertz-Scheraga, 1978 |
| 88 | WERD780103 | Physicochemical property | Free energy change of alpha(Ri) to alpha(Rh) | Wertz-Scheraga, 1978 |
| 89 | WERD780104 | Physicochemical property | Free energy change of epsilon(i) to alpha(Rh) | Wertz-Scheraga, 1978 |
| 90 | YUTK870101 | Physicochemical property | Unfolding Gibbs energy in water, pH 7.0 | Yutani et al., 1987 |
| 91 | YUTK870102 | Physicochemical property | Unfolding Gibbs energy in water, pH 9.0 | Yutani et al., 1987 |
| 92 | YUTK870103 | Physicochemical property | Activation Gibbs energy of unfolding, pH 7.0 | Yutani et al., 1987 |
| 93 | YUTK870104 | Physicochemical property | Activation Gibbs energy of unfolding, pH 9.0 | Yutani et al., 1987 |
| 94 | MUNV940101 | Physicochemical property | Free energy in alpha-helical conformation | Munoz-Serrano, 1994 |
| 95 | MUNV940102 | Physicochemical property | Free energy in alpha-helical region | Munoz-Serrano, 1994 |
| 96 | MUNV940103 | Physicochemical property | Free energy in beta-strand conformation | Munoz-Serrano, 1994 |
| 97 | MUNV940104 | Physicochemical property | Free energy in beta-strand region | Munoz-Serrano, 1994 |
| 98 | MUNV940105 | Physicochemical property | Free energy in beta-strand region | Munoz-Serrano, 1994 |
| 99 | WIMW960101 | Physicochemical property | Free energies of transfer of AcWl-X-LL peptides from bilayer interface to water | Wimley-White, 1996 |
| 100 | MONM990101 | Physicochemical property | Turn propensity scale for transmembrane helices | Monne et al., 1999 |
| 101 | MIYS990101 | Physicochemical property | Relative partition energies derived by the Bethe approximation | Miyazawa-Jernigan, 1999 |
| 102 | MIYS990102 | Physicochemical property | Optimized relative partition energies—method A | Miyazawa-Jernigan, 1999 |
| 103 | MIYS990103 | Physicochemical property | Optimized relative partition energies—method B | Miyazawa-Jernigan, 1999 |
| 104 | MIYS990104 | Physicochemical property | Optimized relative partition energies—method C | Miyazawa-Jernigan, 1999 |
| 105 | MIYS990105 | Physicochemical property | Optimized relative partition energies—method D | Miyazawa-Jernigan, 1999 |
| 106 | HUTJ700102 | Physicochemical property | Absolute entropy | Hutchens, 1970 |
| 107 | HUTJ700103 | Physicochemical property | Entropy of formation | Hutchens, 1970 |
| 108 | BHAR880101 | Physicochemical property | Average flexibility indices | Bhaskaran-Ponnuswamy, 1988 |
| 109 | KARP850101 | Physicochemical property | Flexibility parameter for no rigid neighbors | Karplus-Schulz, 1985 |
| 110 | KARP850102 | Physicochemical property | Flexibility parameter for one rigid neighbor | Karplus-Schulz, 1985 |
| 111 | KARP850103 | Physicochemical property | Flexibility parameter for two rigid neighbors | Karplus-Schulz, 1985 |
| 112 | VINM940101 | Physicochemical property | Normalized flexibility parameters (B-values), average | Vihinen et al., 1994 |
| 113 | VINM940102 | Physicochemical property | Normalized flexibility parameters (B-values) for each residue surrounded by none rigid neighbors | Vihinen et al., 1994 |
| 114 | VINM940103 | Physicochemical property | Normalized flexibility parameters (B-values) for each residue surrounded by one rigid neighbors | Vihinen et al., 1994 |
| 115 | VINM940104 | Physicochemical property | Normalized flexibility parameters (B-values) for each residue surrounded by two rigid neighbors | Vihinen et al., 1994 |
| 116 | PARS000101 | Physicochemical property | p-Values of mesophilic proteins based on the distributions of B-values | Parthasarathy-Murthy, 2000 |
| 117 | PARS000102 | Physicochemical property | p-Values of thermophilic proteins based on the distributions of B-values | Parthasarathy-Murthy, 2000 |
| 118 | FAUJ880101 | Physicochemical property | Graph shape index | Fauchere et al., 1988 |
| 119 | HUTJ700101 | Physicochemical property | Heat capacity | Hutchens, 1970 |
| 120 | PARJ860101 | Physicochemical property | HPLC parameter | Parker et al., 1986 |
| 121 | HOPA770101 | Physicochemical property | Hydration number | Hopfinger, 1971, cited by Charton-Charton , 1982 |
| 122 | KRIW790102 | Physicochemical property | Fraction of site occupied by water | Krigbaum-Komoriya, 1979 |
| 123 | ROBB790101 | Physicochemical property | Hydration free energy | Robson-Osguthorpe, 1979 |
| 124 | WOLR810101 | Physicochemical property | Hydration potential | Wolfenden et al., 1981 |
| 125 | DIGM050101 | Physicochemical property | Hydrostatic pressure asymmetry index, PAI | Di Giulio, 2005 |
| 126 | DESM900102 | Physicochemical property | Average membrane preference: AMP07 | Degli Esposti et al., 1990 |
| 127 | KYTJ820101 | Physicochemical property | Hydropathy index | Kyte-Doolittle, 1982 |
| 128 | ROSM880101 | Physicochemical property | Side chain hydropathy, uncorrected for solvation | Roseman, 1988 |
| 129 | ROSM880102 | Physicochemical property | Side chain hydropathy, corrected for solvation | Roseman, 1988 |
| 130 | ROSM880103 | Physicochemical property | Loss of Side chain hydropathy by helix formation | Roseman, 1988 |
| 131 | NADH010101 | Physicochemical property | Hydropathy scale based on self-information values in the two-state model (5 % accessibility) | Naderi-Manesh et al., 2001 |
| 132 | NADH010102 | Physicochemical property | Hydropathy scale based on self-information values in the two-state model (9 % accessibility) | Naderi-Manesh et al., 2001 |
| 133 | NADH010103 | Physicochemical property | Hydropathy scale based on self-information values in the two-state model (16 % accessibility) | Naderi-Manesh et al., 2001 |
| 134 | NADH010104 | Physicochemical property | Hydropathy scale based on self-information values in the two-state model (20 % accessibility) | Naderi-Manesh et al., 2001 |
| 135 | NADH010105 | Physicochemical property | Hydropathy scale based on self-information values in the two-state model (25 % accessibility) | Naderi-Manesh et al., 2001 |
| 136 | NADH010106 | Physicochemical property | Hydropathy scale based on self-information values in the two-state model (36 % accessibility) | Naderi-Manesh et al., 2001 |
| 137 | NADH010107 | Physicochemical property | Hydropathy scale based on self-information values in the two-state model (50% accessibility) | Naderi-Manesh et al., 2001 |
| 138 | EISD840101 | Physicochemical property | Consensus normalized hydrophobicity scale | Eisenberg, 1984 |
| 139 | EISD860103 | Physicochemical property | Direction of hydrophobic moment | Eisenberg-McLachlan, 1986 |
| 140 | GARJ730101 | Physicochemical property | Partition coefficient | Garel et al., 1973 |
| 141 | HOPT810101 | Physicochemical property | Hydrophilicity value | Hopp-Woods, 1981 |
| 142 | PLIV810101 | Physicochemical property | Partition coefficient | Pliska et al., 1981 |
| 143 | PRAM820101 | Physicochemical property | Intercept in regression analysis | Prabhakaran-Ponnuswamy, 1982 |
| 144 | PRAM820103 | Physicochemical property | Correlation coefficient in regression analysis | Prabhakaran-Ponnuswamy, 1982 |
| 145 | VELV850101 | Physicochemical property | Electron-ion interaction potential | Veljkovic et al., 1985 |
| 146 | ZASB820101 | Physicochemical property | Dependence of partition coefficient on ionic strength | Zaslavsky et al., 1982 |
| 147 | COSI940101 | Physicochemical property | Electron-ion interaction potential values | Cosic, 1994 |
| 148 | KUHL950101 | Physicochemical property | Hydrophilicity scale | Kuhn et al., 1995 |
| 149 | BASU050101 | Physicochemical property | Interactivity scale obtained from the contact matrix | Bastolla et al., 2005 |
| 150 | BASU050102 | Physicochemical property | Interactivity scale obtained by maximizing the mean of correlation coefficient over single-domain globular proteins | Bastolla et al., 2005 |
| 151 | BASU050103 | Physicochemical property | Interactivity scale obtained by maximizing the mean of correlation coefficient over pairs of sequences sharing the TIM barrel fold | Bastolla et al., 2005 |
| 152 | ARGP820101 | Physicochemical property | Hydrophobicity index | Argos et al., 1982 |
| 153 | BULH740102 | Physicochemical property | Apparent partial specific volume | Bull-Breese, 1974 |
| 154 | CIDH920101 | Physicochemical property | Normalized hydrophobicity scales for alpha-proteins | Cid et al., 1992 |
| 155 | CIDH920102 | Physicochemical property | Normalized hydrophobicity scales for beta-proteins | Cid et al., 1992 |
| 156 | CIDH920103 | Physicochemical property | Normalized hydrophobicity scales for alpha+beta-proteins | Cid et al., 1992 |
| 157 | CIDH920104 | Physicochemical property | Normalized hydrophobicity scales for alpha/beta-proteins | Cid et al., 1992 |
| 158 | CIDH920105 | Physicochemical property | Normalized average hydrophobicity scales | Cid et al., 1992 |
| 159 | EISD860102 | Physicochemical property | Atom-based hydrophobic moment | Eisenberg-McLachlan, 1986 |
| 160 | FAUJ830101 | Physicochemical property | Hydrophobic parameter pi | Fauchere-Pliska, 1983 |
| 161 | GOLD730101 | Physicochemical property | Hydrophobicity factor | Goldsack-Chalifoux, 1973 |
| 162 | JOND750101 | Physicochemical property | Hydrophobicity | Jones, 1975 |
| 163 | LEVM760101 | Physicochemical property | Hydrophobic parameter | Levitt, 1976 |
| 164 | MANP780101 | Physicochemical property | Average surrounding hydrophobicity | Manavalan-Ponnuswamy, 1978 |
| 165 | NISK800101 | Physicochemical property | 8 A contact number | Nishikawa-Ooi, 1980 |
| 166 | NISK860101 | Physicochemical property | 14 A contact number | Nishikawa-Ooi, 1986 |
| 167 | PONP800101 | Physicochemical property | Surrounding hydrophobicity in folded form | Ponnuswamy et al., 1980 |
| 168 | PONP800102 | Physicochemical property | Average gain in surrounding hydrophobicity | Ponnuswamy et al., 1980 |
| 169 | PONP800103 | Physicochemical property | Average gain ratio in surrounding hydrophobicity | Ponnuswamy et al., 1980 |
| 170 | PONP800104 | Physicochemical property | Surrounding hydrophobicity in alpha-helix | Ponnuswamy et al., 1980 |
| 171 | PONP800105 | Physicochemical property | Surrounding hydrophobicity in beta-sheet | Ponnuswamy et al., 1980 |
| 172 | PONP800106 | Physicochemical property | Surrounding hydrophobicity in turn | Ponnuswamy et al., 1980 |
| 173 | PRAM900101 | Physicochemical property | Hydrophobicity | Prabhakaran, 1990 |
| 174 | ROSG850101 | Physicochemical property | Mean area buried on transfer | Rose et al., 1985 |
| 175 | ROSG850102 | Physicochemical property | Mean fractional area loss | Rose et al., 1985 |
| 176 | SWER830101 | Physicochemical property | Optimal matching hydrophobicity | Sweet-Eisenberg, 1983 |
| 177 | VENT840101 | Physicochemical property | Bitterness | Venanzi, 1984 |
| 178 | WEBA780101 | Physicochemical property | RF value in high salt chromatography | Weber-Lacey, 1978 |
| 179 | ZIMJ680101 | Physicochemical property | Hydrophobicity | Zimmerman et al., 1968 |
| 180 | ZIMJ680102 | Physicochemical property | Bulkiness | Zimmerman et al., 1968 |
| 181 | ZIMJ680105 | Physicochemical property | RF rank | Zimmerman et al., 1968 |
| 182 | PONP930101 | Physicochemical property | Hydrophobicity scales | Ponnuswamy, 1993 |
| 183 | WILM950101 | Physicochemical property | Hydrophobicity coefficient in RP-HPLC, C18 with 0.1 %TFA/MeCN/H_2_O | Wilce et al., 1995 |
| 184 | WILM950102 | Physicochemical property | Hydrophobicity coefficient in RP-HPLC, C8 with 0.1 %TFA/MeCN/H_2_O | Wilce et al., 1995 |
| 185 | WILM950103 | Physicochemical property | Hydrophobicity coefficient in RP-HPLC, C4 with 0.1 %TFA/MeCN/H_2_O | Wilce et al., 1995 |
| 186 | WILM950104 | Physicochemical property | Hydrophobicity coefficient in RP-HPLC, C18 with 0.1 %TFA/2-PrOH/MeCN/H_2_O | Wilce et al., 1995 |
| 187 | JURD980101 | Physicochemical property | Modified Kyte-Doolittle hydrophobicity scale | Juretic et al., 1998 |
| 188 | WOLR790101 | Physicochemical property | Hydrophobicity index | Wolfenden et al., 1979 |
| 189 | KIDA850101 | Physicochemical property | Hydrophobicity-related index | Kidera et al., 1985 |
| 190 | GUYH850102 | Physicochemical property | Apparent partition energies calculated from Wertz-Scheraga index | Guy, 1985 |
| 191 | GUYH850104 | Physicochemical property | Apparent partition energies calculated from Janin index | Guy, 1985 |
| 192 | GUYH850105 | Physicochemical property | Apparent partition energies calculated from Chothia index | Guy, 1985 |
| 193 | COWR900101 | Physicochemical property | Hydrophobicity index, 3 pH | Cowan-Whittaker, 1990 |
| 194 | BLAS910101 | Physicochemical property | Scaled side chain hydrophobicity values | Black-Mould, 1991 |
| 195 | CASG920101 | Physicochemical property | Hydrophobicity scale from native protein structures | Casari-Sippl, 1992 |
| 196 | CORJ870101 | Physicochemical property | NNEIG index | Cornette et al., 1987 |
| 197 | CORJ870102 | Physicochemical property | SWEIG index | Cornette et al., 1987 |
| 198 | CORJ870103 | Physicochemical property | PRIFT index | Cornette et al., 1987 |
| 199 | CORJ870104 | Physicochemical property | PRILS index | Cornette et al., 1987 |
| 200 | CORJ870105 | Physicochemical property | ALTFT index | Cornette et al., 1987 |
| 201 | CORJ870106 | Physicochemical property | ALTLS index | Cornette et al., 1987 |
| 202 | CORJ870107 | Physicochemical property | TOTFT index | Cornette et al., 1987 |
| 203 | CORJ870108 | Physicochemical property | TOTLS index | Cornette et al., 1987 |
| 204 | ENGD860101 | Physicochemical property | Hydrophobicity index | Engelman et al., 1986 |
| 205 | FASG890101 | Physicochemical property | Hydrophobicity index | Fasman, 1989 |
| 206 | ZIMJ680104 | Physicochemical property | Isoelectric point | Zimmerman et al., 1968 |
| 207 | FASG760102 | Physicochemical property | Melting point | Fasman, 1976 |
| 208 | DAYM780201 | Physicochemical property | Relative mutability | Dayhoff et al., 1978b |
| 209 | JOND920102 | Physicochemical property | Relative mutability | Jones et al., 1992 |
| 210 | FASG760104 | Physicochemical property | pK-N | Fasman, 1976 |
| 211 | FASG760105 | Physicochemical property | pK-C | Fasman, 1976 |
| 212 | FAUJ880113 | Physicochemical property | pK-a(RCOOH | Fauchere et al., 1988 |
| 213 | JOND750102 | Physicochemical property | pK (-COOH) | Jones, 1975 |
| 214 | CHAM820101 | Physicochemical property | Polarizability parameter | Charton-Charton, 1982 |
| 215 | GRAR740102 | Physicochemical property | Polarity | Grantham, 1974 |
| 216 | KHAG800101 | Physicochemical property | The Kerr-constant increments | Khanarian-Moore, 1980 |
| 217 | RADA880108 | Physicochemical property | Mean polarity | Radzicka-Wolfenden, 1988 |
| 218 | WOEC730101 | Physicochemical property | Polar requirement | Woese, 1973 |
| 219 | ZIMJ680103 | Physicochemical property | Polarity | Zimmerman et al., 1968 |
| 220 | ONEK900101 | Physicochemical property | Delta G values for the peptides extrapolated to 0 M urea | O'Neil-DeGrado, 1990 |
| 221 | MITS020101 | Physicochemical property | Amphiphilicity index | Mitaku et al., 2002 |
| 222 | MCMT640101 | Physicochemical property | Refractivity | McMeekin et al., 1964, cited by Jones, 1975 |
| 223 | BROC820101 | Physicochemical property | Retention coefficient in TFA | Browne et al., 1982 |
| 224 | BROC820102 | Physicochemical property | Retention coefficient in HFBA | Browne et al., 1982 |
| 225 | MEEJ800101 | Physicochemical property | Retention coefficient in HPLC, pH 7.4 | Meek, 1980 |
| 226 | MEEJ800102 | Physicochemical property | Retention coefficient in HPLC, pH 2.1 | Meek, 1980 |
| 227 | MEEJ810101 | Physicochemical property | Retention coefficient in NaClO_4_ | Meek-Rossetti, 1981 |
| 228 | MEEJ810102 | Physicochemical property | Retention coefficient in NaH_2_PO_4_ | Meek-Rossetti, 1981 |
| 229 | GUOD860101 | Physicochemical property | Retention coefficient at pH 2 | Guo et al., 1986 |
| 230 | FASG760103 | Physicochemical property | Optical rotation | Fasman, 1976 |
| 231 | DAWD720101 | Physicochemical property | Size | Dawson, 1972 |
| 232 | PRAM820102 | Physicochemical property | Slope in regression analysis × 1.00 ×10^1^ | Prabhakaran-Ponnuswamy, 1982 |
| 233 | TAKK010101 | Physicochemical property | Side-chain contribution to protein stability (kJ/mol) | Takano-Yutani, 2001 |
| 234 | ZHOH040101 | Physicochemical property | The stability scale from the knowledge-based atom-atom potential | Zhou-Zhou, 2004 |
| 235 | ZHOH040102 | Physicochemical property | The relative stability scale extracted from mutation experiments | Zhou-Zhou, 2004 |
| 236 | BUNA790103 | Physicochemical property | Spin-spin coupling constants 3JHalpha-NH | Bundi-Wuthrich, 1979 |
| 237 | CHAM810101 | Physicochemical property | Steric parameter | Charton, 1981 |
| 238 | FAUJ880102 | Physicochemical property | Smoothed upsilon steric parameter | Fauchere et al., 1988 |
| 239 | FAUJ880104 | Physicochemical property | STERIMOL length of the side chain | Fauchere et al., 1988 |
| 240 | FAUJ880105 | Physicochemical property | STERIMOL minimum width of the side chain | Fauchere et al., 1988 |
| 241 | FAUJ880106 | Physicochemical property | STERIMOL maximum width of the side chain | Fauchere et al., 1988 |
| 242 | KUMS000101 | Physicochemical property | Distribution of amino acid residues in the 18 non-redundant families of thermophilic proteins | Kumar et al., 2000 |
| 243 | KUMS000102 | Physicochemical property | Distribution of amino acid residues in the 18 non-redundant families of mesophilic proteins | Kumar et al., 2000 |
| 244 | KUMS000103 | Physicochemical property | Distribution of amino acid residues in the alpha-helices in thermophilic proteins | Kumar et al., 2000 |
| 245 | KUMS000104 | Physicochemical property | Distribution of amino acid residues in the alpha-helices in mesophilic proteins | Kumar et al., 2000 |
| 246 | RACS820113 | Physicochemical property | Value of theta(i) | Rackovsky-Scheraga, 1982 |
| 247 | RACS820114 | Physicochemical property | Value of theta(i-1) | Rackovsky-Scheraga, 1982 |
| 248 | LEVM760106 | Physicochemical property | van der Waals parameter R0 | Levitt, 1976 |
| 249 | LEVM760107 | Physicochemical property | van der Waals parameter epsilon | Levitt, 1976 |
| 250 | BIGC670101 | Physicochemical property | Residue volume | Bigelow, 1967 |
| 251 | COHE430101 | Physicochemical property | Partial specific volume | Cohn-Edsall, 1943 |
| 252 | FAUJ880103 | Physicochemical property | Normalized van der Waals volume | Fauchere et al., 1988 |
| 253 | GOLD730102 | Physicochemical property | Residue volume | Goldsack-Chalifoux, 1973 |
| 254 | GRAR740103 | Physicochemical property | Volume | Grantham, 1974 |
| 255 | TSAJ990101 | Physicochemical property | Volumes including the crystallographic waters using the ProtOr | Tsai et al., 1999 |
| 256 | TSAJ990102 | Physicochemical property | Volumes not including the crystallographic waters using the ProtOr | Tsai et al., 1999 |
| 257 | HARY940101 | Physicochemical property | Mean volumes of residues buried in protein interiors | Harpaz et al., 1994 |
| 258 | FASG760101 | Physicochemical property | Molecular weight | Fasman, 1976 |
| 259 | BEGF750102 | Second structure | Conformational parameter of beta-structure | Beghin-Dirkx, 1975 |
| 260 | CHAM830102 | Second structure | A parameter defined from the residuals obtained from the best correlation of the Chou-Fasman parameter of beta-sheet | Charton-Charton, 1983 |
| 261 | CHOP780202 | Second structure | Normalized frequency of beta-sheet | Chou-Fasman, 1978b |
| 262 | CHOP780208 | Second structure | Normalized frequency of N-terminal beta-sheet | Chou-Fasman, 1978b |
| 263 | CHOP780209 | Second structure | Normalized frequency of C-terminal beta-sheet | Chou-Fasman, 1978b |
| 264 | CHOP780210 | Second structure | Normalized frequency of N-terminal non beta region | Chou-Fasman, 1978b |
| 265 | CHOP780211 | Second structure | Normalized frequency of C-terminal non beta region | Chou-Fasman, 1978b |
| 266 | CRAJ730102 | Second structure | Normalized frequency of beta-sheet | Crawford et al., 1973 |
| 267 | KANM800102 | Second structure | Average relative probability of beta-sheet | Kanehisa-Tsong, 1980 |
| 268 | KANM800104 | Second structure | Average relative probability of inner beta-sheet | Kanehisa-Tsong, 1980 |
| 269 | LEVM780102 | Second structure | Normalized frequency of beta-sheet, with weights | Levitt, 1978 |
| 270 | LEVM780105 | Second structure | Normalized frequency of beta-sheet, unweighted | Levitt, 1978 |
| 271 | LEWP710101 | Second structure | Frequency of occurrence in beta-bends | Lewis et al., 1971 |
| 272 | NAGK730102 | Second structure | Normalized frequency of beta-structure | Nagano, 1973 |
| 273 | PALJ810103 | Second structure | Normalized frequency of beta-sheet from LG | Palau et al., 1981 |
| 274 | PALJ810104 | Second structure | Normalized frequency of beta-sheet from CF | Palau et al., 1981 |
| 275 | PALJ810110 | Second structure | Normalized frequency of beta-sheet in all-beta class | Palau et al., 1981 |
| 276 | PALJ810111 | Second structure | Normalized frequency of beta-sheet in alpha+beta class | Palau et al., 1981 |
| 277 | PALJ810112 | Second structure | Normalized frequency of beta-sheet in alpha/beta class | Palau et al., 1981 |
| 278 | PRAM900103 | Second structure | Relative frequency in beta-sheet | Prabhakaran, 1990 |
| 279 | QIAN880114 | Second structure | Weights for beta-sheet at the window position of –6 | Qian-Sejnowski, 1988 |
| 280 | QIAN880115 | Second structure | Weights for beta-sheet at the window position of –5 | Qian-Sejnowski, 1988 |
| 281 | QIAN880116 | Second structure | Weights for beta-sheet at the window position of –4 | Qian-Sejnowski, 1988 |
| 282 | QIAN880117 | Second structure | Weights for beta-sheet at the window position of –3 | Qian-Sejnowski, 1988 |
| 283 | QIAN880118 | Second structure | Weights for beta-sheet at the window position of –2 | Qian-Sejnowski, 1988 |
| 284 | QIAN880119 | Second structure | Weights for beta-sheet at the window position of –1 | Qian-Sejnowski, 1988 |
| 285 | QIAN880120 | Second structure | Weights for beta-sheet at the window position of 0 | Qian-Sejnowski, 1988 |
| 286 | QIAN880121 | Second structure | Weights for beta-sheet at the window position of 1 | Qian-Sejnowski, 1988 |
| 287 | QIAN880122 | Second structure | Weights for beta-sheet at the window position of 2 | Qian-Sejnowski, 1988 |
| 288 | QIAN880123 | Second structure | Weights for beta-sheet at the window position of 3 | Qian-Sejnowski, 1988 |
| 289 | QIAN880124 | Second structure | Weights for beta-sheet at the window position of 4 | Qian-Sejnowski, 1988 |
| 290 | QIAN880125 | Second structure | Weights for beta-sheet at the window position of 5 | Qian-Sejnowski, 1988 |
| 291 | QIAN880126 | Second structure | Weights for beta-sheet at the window position of 6 | Qian-Sejnowski, 1988 |
| 292 | KIMC930101 | Second structure | Thermodynamic beta sheet propensity | Kim-Berg, 1993 |
| 293 | KOEP990102 | Second structure | Beta-sheet propensity derived from designed sequences | Koehl-Levitt, 1999 |
| 294 | GEIM800105 | Second structure | Beta-strand indices | Geisow-Roberts, 1980 |
| 295 | GEIM800106 | Second structure | Beta-strand indices for beta-proteins | Geisow-Roberts, 1980 |
| 296 | GEIM800107 | Second structure | Beta-strand indices for alpha/beta-proteins | Geisow-Roberts, 1980 |
| 297 | LIFS790101 | Second structure | Conformational preference for all beta-strands | Lifson-Sander, 1979 |
| 298 | LIFS790102 | Second structure | Conformational preference for parallel beta-strands | Lifson-Sander, 1979 |
| 299 | LIFS790103 | Second structure | Conformational preference for antiparallel beta-strands | Lifson-Sander, 1979 |
| 300 | OOBM850101 | Second structure | Optimized beta-structure-coil equilibrium constant | Oobatake et al., 1985 |
| 301 | PTIO830102 | Second structure | Beta-coil equilibrium constant | Ptitsyn-Finkelstein, 1983 |
| 302 | BEGF750103 | Second structure | Conformational parameter of beta-turn | Beghin-Dirkx, 1975 |
| 303 | CHAM830101 | Second structure | The Chou-Fasman parameter of the coil conformation | Charton-Charton, 1983 |
| 304 | ISOY800108 | Second structure | Normalized relative frequency of coil | Isogai et al., 1980 |
| 305 | NAGK730103 | Second structure | Normalized frequency of coil | Nagano, 1973 |
| 306 | QIAN880127 | Second structure | Weights for coil at the window position of –6 | Qian-Sejnowski, 1988 |
| 307 | QIAN880128 | Second structure | Weights for coil at the window position of –5 | Qian-Sejnowski, 1988 |
| 308 | QIAN880129 | Second structure | Weights for coil at the window position of –4 | Qian-Sejnowski, 1988 |
| 309 | QIAN880130 | Second structure | Weights for coil at the window position of –3 | Qian-Sejnowski, 1988 |
| 310 | QIAN880131 | Second structure | Weights for coil at the window position of –2 | Qian-Sejnowski, 1988 |
| 311 | QIAN880132 | Second structure | Weights for coil at the window position of –1 | Qian-Sejnowski, 1988 |
| 312 | QIAN880133 | Second structure | Weights for coil at the window position of 0 | Qian-Sejnowski, 1988 |
| 313 | QIAN880134 | Second structure | Weights for coil at the window position of 1 | Qian-Sejnowski, 1988 |
| 314 | QIAN880135 | Second structure | Weights for coil at the window position of 2 | Qian-Sejnowski, 1988 |
| 315 | QIAN880136 | Second structure | Weights for coil at the window position of 3 | Qian-Sejnowski, 1988 |
| 316 | QIAN880137 | Second structure | Weights for coil at the window position of 4 | Qian-Sejnowski, 1988 |
| 317 | QIAN880138 | Second structure | Weights for coil at the window position of 5 | Qian-Sejnowski, 1988 |
| 318 | QIAN880139 | Second structure | Weights for coil at the window position of 6 | Qian-Sejnowski, 1988 |
| 319 | ROBB760112 | Second structure | Information measure for coil | Robson-Suzuki, 1976 |
| 320 | TANS770109 | Second structure | Normalized frequency of coil | Tanaka-Scheraga, 1977 |
| 321 | BURA740102 | Second structure | Normalized frequency of extended structure | Burgess et al., 1974 |
| 322 | CHOC760103 | Second structure | Proportion of residues 95 % buried | Chothia, 1976 |
| 323 | CHOC760104 | Second structure | Proportion of residues 100 % buried | Chothia, 1976 |
| 324 | ISOY800102 | Second structure | Normalized relative frequency of extended structure | Isogai et al., 1980 |
| 325 | ISOY800103 | Second structure | Normalized relative frequency of bend | Isogai et al., 1980 |
| 326 | ISOY800104 | Second structure | Normalized relative frequency of bend R | Isogai et al., 1980 |
| 327 | ISOY800105 | Second structure | Normalized relative frequency of bend S | Isogai et al., 1980 |
| 328 | ISOY800107 | Second structure | Normalized relative frequency of double bend | Isogai et al., 1980 |
| 329 | JANJ780102 | Second structure | Percentage of buried residues | Janin et al., 1978 |
| 330 | JANJ780103 | Second structure | Percentage of exposed residues | Janin et al., 1978 |
| 331 | MAXF760102 | Second structure | Normalized frequency of extended structure | Maxfield-Scheraga, 1976 |
| 332 | MAXF760103 | Second structure | Normalized frequency of zeta R | Maxfield-Scheraga, 1976 |
| 333 | MAXF760105 | Second structure | Normalized frequency of zeta L | Maxfield-Scheraga, 1976 |
| 334 | TANS770103 | Second structure | Normalized frequency of extended structure | Tanaka-Scheraga, 1977 |
| 335 | TANS770104 | Second structure | Normalized frequency of chain reversal R | Tanaka-Scheraga, 1977 |
| 336 | TANS770105 | Second structure | Normalized frequency of chain reversal S | Tanaka-Scheraga, 1977 |
| 337 | TANS770106 | Second structure | Normalized frequency of chain reversal D | Tanaka-Scheraga, 1977 |
| 338 | TANS770108 | Second structure | Normalized frequency of zeta R | Tanaka-Scheraga, 1977 |
| 339 | TANS770110 | Second structure | Normalized frequency of chain reversal | Tanaka-Scheraga, 1977 |
| 340 | WERD780101 | Second structure | Propensity to be buried inside | Wertz-Scheraga, 1978 |
| 341 | JOND920101 | Second structure | Relative frequency of occurrence | Jones et al., 1992 |
| 342 | ARGP820102 | Second structure | Signal sequence helical potential | Argos et al., 1982 |
| 343 | BEGF750101 | Second structure | Conformational parameter of inner helix | Beghin-Dirkx, 1975 |
| 344 | BURA740101 | Second structure | Normalized frequency of alpha-helix | Burgess et al., 1974 |
| 345 | CHOP780201 | Second structure | Normalized frequency of alpha-helix | Chou-Fasman, 1978b |
| 346 | CHOP780204 | Second structure | Normalized frequency of N-terminal helix | Chou-Fasman, 1978b |
| 347 | CHOP780205 | Second structure | Normalized frequency of C-terminal helix | Chou-Fasman, 1978b |
| 348 | CHOP780206 | Second structure | Normalized frequency of N-terminal non helical region | Chou-Fasman, 1978b |
| 349 | CHOP780207 | Second structure | Normalized frequency of C-terminal non helical region | Chou-Fasman, 1978b |
| 350 | CRAJ730101 | Second structure | Normalized frequency of middle helix | Crawford et al., 1973 |
| 351 | FINA910101 | Second structure | Helix initiation parameter at position i–1 | Finkelstein et al., 1991 |
| 352 | FINA910102 | Second structure | Helix initiation parameter at position i,i+1,i+2 | Finkelstein et al., 1991 |
| 353 | FINA910103 | Second structure | Helix termination parameter at position j–2,j–1,j | Finkelstein et al., 1991 |
| 354 | FINA910104 | Second structure | Helix termination parameter at position j+1 | Finkelstein et al., 1991 |
| 355 | GEIM800101 | Second structure | Alpha-helix indices | Geisow-Roberts, 1980 |
| 356 | GEIM800102 | Second structure | Alpha-helix indices for alpha-proteins | Geisow-Roberts, 1980 |
| 357 | GEIM800103 | Second structure | Alpha-helix indices for beta-proteins | Geisow-Roberts, 1980 |
| 358 | GEIM800104 | Second structure | Alpha-helix indices for alpha/beta-proteins | Geisow-Roberts, 1980 |
| 359 | ISOY800101 | Second structure | Normalized relative frequency of alpha-helix | Isogai et al., 1980 |
| 360 | ISOY800106 | Second structure | Normalized relative frequency of helix end | Isogai et al., 1980 |
| 361 | KANM800101 | Second structure | Average relative probability of helix | Kanehisa-Tsong, 1980 |
| 362 | KANM800103 | Second structure | Average relative probability of inner helix | Kanehisa-Tsong, 1980 |
| 363 | LEVM780101 | Second structure | Normalized frequency of alpha-helix, with weights | Levitt, 1978 |
| 364 | LEVM780104 | Second structure | Normalized frequency of alpha-helix, unweighted | Levitt, 1978 |
| 365 | MAXF760101 | Second structure | Normalized frequency of alpha-helix | Maxfield-Scheraga, 1976 |
| 366 | MAXF760104 | Second structure | Normalized frequency of left-handed alpha-helix | Maxfield-Scheraga, 1976 |
| 367 | MAXF760106 | Second structure | Normalized frequency of alpha region | Maxfield-Scheraga, 1976 |
| 368 | NAGK730101 | Second structure | Normalized frequency of alpha-helix | Nagano, 1973 |
| 369 | PALJ810101 | Second structure | Normalized frequency of alpha-helix from LG | Palau et al., 1981 |
| 370 | PALJ810102 | Second structure | Normalized frequency of alpha-helix from CF | Palau et al., 1981 |
| 371 | PALJ810107 | Second structure | Normalized frequency of alpha-helix in all-alpha class | Palau et al., 1981 |
| 372 | PALJ810108 | Second structure | Normalized frequency of alpha-helix in alpha+beta class | Palau et al., 1981 |
| 373 | PALJ810109 | Second structure | Normalized frequency of alpha-helix in alpha/beta class | Palau et al., 1981 |
| 374 | PRAM900102 | Second structure | Relative frequency in alpha-helix | Prabhakaran, 1990 |
| 375 | QIAN880101 | Second structure | Weights for alpha-helix at the window position of –6 | Qian-Sejnowski, 1988 |
| 376 | QIAN880102 | Second structure | Weights for alpha-helix at the window position of –5 | Qian-Sejnowski, 1988 |
| 377 | QIAN880103 | Second structure | Weights for alpha-helix at the window position of –4 | Qian-Sejnowski, 1988 |
| 378 | QIAN880104 | Second structure | Weights for alpha-helix at the window position of –3 | Qian-Sejnowski, 1988 |
| 379 | QIAN880105 | Second structure | Weights for alpha-helix at the window position of –2 | Qian-Sejnowski, 1988 |
| 380 | QIAN880106 | Second structure | Weights for alpha-helix at the window position of –1 | Qian-Sejnowski, 1988 |
| 381 | QIAN880107 | Second structure | Weights for alpha-helix at the window position of 0 | Qian-Sejnowski, 1988 |
| 382 | QIAN880108 | Second structure | Weights for alpha-helix at the window position of 1 | Qian-Sejnowski, 1988 |
| 383 | QIAN880109 | Second structure | Weights for alpha-helix at the window position of 2 | Qian-Sejnowski, 1988 |
| 384 | QIAN880110 | Second structure | Weights for alpha-helix at the window position of 3 | Qian-Sejnowski, 1988 |
| 385 | QIAN880111 | Second structure | Weights for alpha-helix at the window position of 4 | Qian-Sejnowski, 1988 |
| 386 | QIAN880112 | Second structure | Weights for alpha-helix at the window position of 5 | Qian-Sejnowski, 1988 |
| 387 | QIAN880113 | Second structure | Weights for alpha-helix at the window position of 6 | Qian-Sejnowski, 1988 |
| 388 | ROBB760101 | Second structure | Information measure for alpha-helix | Robson-Suzuki, 1976 |
| 389 | ROBB760102 | Second structure | Information measure for N-terminal helix | Robson-Suzuki, 1976 |
| 390 | ROBB760103 | Second structure | Information measure for middle helix | Robson-Suzuki, 1976 |
| 391 | ROBB760104 | Second structure | Information measure for C-terminal helix | Robson-Suzuki, 1976 |
| 392 | TANS770101 | Second structure | Normalized frequency of alpha-helix | Tanaka-Scheraga, 1977 |
| 393 | TANS770102 | Second structure | Normalized frequency of isolated helix | Tanaka-Scheraga, 1977 |
| 394 | TANS770107 | Second structure | Normalized frequency of left-handed helix | Tanaka-Scheraga, 1977 |
| 395 | AURR980101 | Second structure | Normalized positional residue frequency at helix termini N4' | Aurora-Rose, 1998 |
| 396 | AURR980102 | Second structure | Normalized positional residue frequency at helix termini N"' | Aurora-Rose, 1998 |
| 397 | AURR980103 | Second structure | Normalized positional residue frequency at helix termini N" | Aurora-Rose, 1998 |
| 398 | AURR980104 | Second structure | Normalized positional residue frequency at helix termini N' | Aurora-Rose, 1998 |
| 399 | AURR980105 | Second structure | Normalized positional residue frequency at helix termini Nc | Aurora-Rose, 1998 |
| 400 | AURR980106 | Second structure | Normalized positional residue frequency at helix termini N1 | Aurora-Rose, 1998 |
| 401 | AURR980107 | Second structure | Normalized positional residue frequency at helix termini N2 | Aurora-Rose, 1998 |
| 402 | AURR980108 | Second structure | Normalized positional residue frequency at helix termini N3 | Aurora-Rose, 1998 |
| 403 | AURR980109 | Second structure | Normalized positional residue frequency at helix termini N4 | Aurora-Rose, 1998 |
| 404 | AURR980110 | Second structure | Normalized positional residue frequency at helix termini N5 | Aurora-Rose, 1998 |
| 405 | AURR980111 | Second structure | Normalized positional residue frequency at helix termini C5 | Aurora-Rose, 1998 |
| 406 | AURR980112 | Second structure | Normalized positional residue frequency at helix termini C4 | Aurora-Rose, 1998 |
| 407 | AURR980113 | Second structure | Normalized positional residue frequency at helix termini C3 | Aurora-Rose, 1998 |
| 408 | AURR980114 | Second structure | Normalized positional residue frequency at helix termini C2 | Aurora-Rose, 1998 |
| 409 | AURR980115 | Second structure | Normalized positional residue frequency at helix termini C1 | Aurora-Rose, 1998 |
| 410 | AURR980116 | Second structure | Normalized positional residue frequency at helix termini Cc | Aurora-Rose, 1998 |
| 411 | AURR980117 | Second structure | Normalized positional residue frequency at helix termini C' | Aurora-Rose, 1998 |
| 412 | AURR980118 | Second structure | Normalized positional residue frequency at helix termini C" | Aurora-Rose, 1998 |
| 413 | AURR980119 | Second structure | Normalized positional residue frequency at helix termini C"' | Aurora-Rose, 1998 |
| 414 | AURR980120 | Second structure | Normalized positional residue frequency at helix termini C4' | Aurora-Rose, 1998 |
| 415 | ONEK900102 | Second structure | Helix formation parameters (delta delta G) | O'Neil-DeGrado, 1990 |
| 416 | BLAM930101 | Second structure | Alpha helix propensity of position 44 in T4 lysozyme | Blaber et al., 1993 |
| 417 | FODM020101 | Second structure | Propensity of amino acids within pi-helices | Fodje-Al-Karadaghi, 2002 |
| 418 | MONM990201 | Second structure | Averaged turn propensities in a transmembrane helix | Monne et al., 1999 |
| 419 | KOEP990101 | Second structure | Alpha-helix propensity derived from designed sequences | Koehl-Levitt, 1999 |
| 420 | PUNT030101 | Second structure | Knowledge-based membrane-propensity scale from 1D_Helix in MPtopo databases | Punta-Maritan, 2003 |
| 421 | PUNT030102 | Second structure | Knowledge-based membrane-propensity scale from 3D_Helix in MPtopo databases | Punta-Maritan, 2003 |
| 422 | GEOR030108 | Second structure | Linker propensity from helical (annotated by DSSP dataset) | Punta-Maritan, 2003 |
| 423 | JACR890101 | Second structure | Weights from the IFH scale | Jacobs-White, 1989 |
| 424 | FINA770101 | Second structure | Helix-coil equilibrium constant | Finkelstein-Ptitsyn, 1977 |
| 425 | PTIO830101 | Second structure | Helix-coil equilibrium constant | Ptitsyn-Finkelstein, 1983 |
| 426 | RICJ880101 | Second structure | Relative preference value at N" | Richardson-Richardson, 1988 |
| 427 | RICJ880102 | Second structure | Relative preference value at N' | Richardson-Richardson, 1988 |
| 428 | RICJ880103 | Second structure | Relative preference value at N-cap | Richardson-Richardson, 1988 |
| 429 | RICJ880104 | Second structure | Relative preference value at N1 | Richardson-Richardson, 1988 |
| 430 | RICJ880105 | Second structure | Relative preference value at N2 | Richardson-Richardson, 1988 |
| 431 | RICJ880106 | Second structure | Relative preference value at N3 | Richardson-Richardson, 1988 |
| 432 | RICJ880107 | Second structure | Relative preference value at N4 | Richardson-Richardson, 1988 |
| 433 | RICJ880108 | Second structure | Relative preference value at N5 | Richardson-Richardson, 1988 |
| 434 | RICJ880109 | Second structure | Relative preference value at Mid | Richardson-Richardson, 1988 |
| 435 | RICJ880110 | Second structure | Relative preference value at C5 | Richardson-Richardson, 1988 |
| 436 | RICJ880111 | Second structure | Relative preference value at C4 | Richardson-Richardson, 1988 |
| 437 | RICJ880112 | Second structure | Relative preference value at C3 | Richardson-Richardson, 1988 |
| 438 | RICJ880113 | Second structure | Relative preference value at C2 | Richardson-Richardson, 1988 |
| 439 | RICJ880114 | Second structure | Relative preference value at C1 | Richardson-Richardson, 1988 |
| 440 | RICJ880115 | Second structure | Relative preference value at C-cap | Richardson-Richardson, 1988 |
| 441 | RICJ880116 | Second structure | Relative preference value at C' | Richardson-Richardson, 1988 |
| 442 | RICJ880117 | Second structure | Relative preference value at C" | Richardson-Richardson, 1988 |
| 443 | ARGP820103 | Second structure | Membrane-buried preference parameters | Argos et al., 1982 |
| 444 | DESM900101 | Second structure | Membrane preference for cytochrome b: MPH89 | Degli Esposti et al., 1990 |
| 445 | SUYM030101 | Second structure | Linker propensity index | Suyama-Ohara, 2003 |
| 446 | GEOR030101 | Second structure | Linker propensity from all dataset | George-Heringa, 2003 |
| 447 | GEOR030102 | Second structure | Linker propensity from 1-linker dataset | George-Heringa, 2003 |
| 448 | GEOR030103 | Second structure | Linker propensity from 2-linker dataset | George-Heringa, 2003 |
| 449 | GEOR030104 | Second structure | Linker propensity from 3-linker dataset | George-Heringa, 2003 |
| 450 | GEOR030105 | Second structure | Linker propensity from small dataset (linker length is less than six residues) | George-Heringa, 2003 |
| 451 | GEOR030106 | Second structure | Linker propensity from medium dataset (linker length is between six and 14 residues) | George-Heringa, 2003 |
| 452 | GEOR030107 | Second structure | Linker propensity from long dataset (linker length is greater than 14 residues) | George-Heringa, 2003 |
| 453 | GEOR030109 | Second structure | Linker propensity from non-helical (annotated by DSSP dataset) | George-Heringa, 2003 |
| 454 | BAEK050101 | Second structure | Linker index | Bae et al., 2005 |
| 455 | CHOC750101 | Second structure | Average volume of buried residue | Chothia, 1975 |
| 456 | PONP800108 | Second structure | Average number of surrounding residues | Ponnuswamy et al., 1980 |
| 457 | VASM830101 | Second structure | Relative population of conformational state A | Vasquez et al., 1983 |
| 458 | VASM830102 | Second structure | Relative population of conformational state C | Vasquez et al., 1983 |
| 459 | VASM830103 | Second structure | Relative population of conformational state E | Vasquez et al., 1983 |
| 460 | PONJ960101 | Second structure | Average volumes of residues | Pontius et al., 1996 |
| 461 | OLSK800101 | Second structure | Average internal preferences | Olsen, 1980 |
| 462 | ANDN920101 | Second structure | alpha-CH chemical shifts | Andersen et al., 1992 |
| 463 | BUNA790101 | Second structure | alpha-NH chemical shifts | Bundi-Wuthrich, 1979 |
| 464 | BUNA790102 | Second structure | alpha-CH chemical shifts | Bundi-Wuthrich, 1979 |
| 465 | GEIM800108 | Second structure | Aperiodic indices | Geisow-Roberts, 1980 |
| 466 | GEIM800109 | Second structure | Aperiodic indices for alpha-proteins | Geisow-Roberts, 1980 |
| 467 | GEIM800110 | Second structure | Aperiodic indices for beta-proteins | Geisow-Roberts, 1980 |
| 468 | GEIM800111 | Second structure | Aperiodic indices for alpha/beta-proteins | Geisow-Roberts, 1980 |
| 469 | MEIH800101 | Second structure | Average reduced distance for C-alpha | Meirovitch et al., 1980 |
| 470 | RACS770101 | Second structure | Average reduced distance for C-alpha | Rackovsky-Scheraga, 1977 |
| 471 | RACS820101 | Second structure | Average relative fractional occurrence in A0(i) | Rackovsky-Scheraga, 1982 |
| 472 | RACS820102 | Second structure | Average relative fractional occurrence in AR(i) | Rackovsky-Scheraga, 1982 |
| 473 | RACS820103 | Second structure | Average relative fractional occurrence in AL(i) | Rackovsky-Scheraga, 1982 |
| 474 | RACS820104 | Second structure | Average relative fractional occurrence in EL(i) | Rackovsky-Scheraga, 1982 |
| 475 | RACS820105 | Second structure | Average relative fractional occurrence in E0(i) | Rackovsky-Scheraga, 1982 |
| 476 | RACS820106 | Second structure | Average relative fractional occurrence in ER(i) | Rackovsky-Scheraga, 1982 |
| 477 | RACS820107 | Second structure | Average relative fractional occurrence in A0(i-1) | Rackovsky-Scheraga, 1982 |
| 478 | RACS820108 | Second structure | Average relative fractional occurrence in AR(i-1) | Rackovsky-Scheraga, 1982 |
| 479 | RACS820109 | Second structure | Average relative fractional occurrence in AL(i-1) | Rackovsky-Scheraga, 1982 |
| 480 | RACS820110 | Second structure | Average relative fractional occurrence in EL(i-1) | Rackovsky-Scheraga, 1982 |
| 481 | RACS820111 | Second structure | Average relative fractional occurrence in E0(i-1) | Rackovsky-Scheraga, 1982 |
| 482 | RACS820112 | Second structure | Average relative fractional occurrence in ER(i-1) | Rackovsky-Scheraga, 1982 |
| 483 | ROBB760105 | Second structure | Information measure for extended | Robson-Suzuki, 1976 |
| 484 | ROBB760106 | Second structure | Information measure for pleated-sheet | Robson-Suzuki, 1976 |
| 485 | ROBB760107 | Second structure | Information measure for extended without H-bond | Robson-Suzuki, 1976 |
| 486 | ROBB760113 | Second structure | Information measure for loop | Robson-Suzuki, 1976 |
| 487 | CHAM830103 | Second structure | The number of atoms in the side chain labelled 1+1 | Charton-Charton, 1983 |
| 488 | CHAM830104 | Second structure | The number of atoms in the side chain labelled 2+1 | Charton-Charton, 1983 |
| 489 | CHAM830105 | Second structure | The number of atoms in the side chain labelled 3+1 | Charton-Charton, 1983 |
| 490 | CHAM830106 | Second structure | The number of bonds in the longest chain | Charton-Charton, 1983 |
| 491 | FAUJ880107 | Second structure | N.m.r. chemical shift of alpha-carbon | Fauchere et al., 1988 |
| 492 | FAUJ880109 | Second structure | Number of hydrogen bond donors | Fauchere et al., 1988 |
| 493 | FAUJ880110 | Second structure | Number of full nonbonding orbitals | Fauchere et al., 1988 |
| 494 | KRIW710101 | Second structure | Side chain interaction parameter | Krigbaum-Rubin, 1971 |
| 495 | KRIW790101 | Second structure | Side chain interaction parameter | Krigbaum-Komoriya, 1979 |
| 496 | KRIW790103 | Second structure | Side chain volume | Krigbaum-Komoriya, 1979 |
| 497 | LEVM760102 | Second structure | Distance between C-alpha and centroid of side chain | Levitt, 1976 |
| 498 | LEVM760103 | Second structure | Side chain angle theta(AAR) | Levitt, 1976 |
| 499 | LEVM760104 | Second structure | Side chain torsion angle phi(AAAR) | Levitt, 1976 |
| 500 | LEVM760105 | Second structure | Radius of gyration of side chain | Levitt, 1976 |
| 501 | MEIH800102 | Second structure | Average reduced distance for side chain | Meirovitch et al., 1980 |
| 502 | MEIH800103 | Second structure | Average side chain orientation angle | Meirovitch et al., 1980 |
| 503 | OOBM850105 | Second structure | Optimized side chain interaction parameter | Oobatake et al., 1985 |
| 504 | RACS770102 | Second structure | Average reduced distance for side chain | Rackovsky-Scheraga, 1977 |
| 505 | RACS770103 | Second structure | Side chain orientational preference | Rackovsky-Scheraga, 1977 |
| 506 | WARP780101 | Second structure | Average interactions per side chain atom | Warme-Morgan, 1978 |
| 507 | ZHOH040103 | Second structure | Buriability | Zhou-Zhou, 2004 |
| 508 | CHOP780101 | Second structure | Normalized frequency of beta-turn | Chou-Fasman, 1978a |
| 509 | CHOP780203 | Second structure | Normalized frequency of beta-turn | Chou-Fasman, 1978b |
| 510 | CHOP780212 | Second structure | Frequency of the 1st residue in turn | Chou-Fasman, 1978b |
| 511 | CHOP780213 | Second structure | Frequency of the 2nd residue in turn | Chou-Fasman, 1978b |
| 512 | CHOP780214 | Second structure | Frequency of the 3rd residue in turn | Chou-Fasman, 1978b |
| 513 | CHOP780215 | Second structure | Frequency of the 4th residue in turn | Chou-Fasman, 1978b |
| 514 | CHOP780216 | Second structure | Normalized frequency of the 2nd and 3rd residues in turn | Chou-Fasman, 1978b |
| 515 | CRAJ730103 | Second structure | Normalized frequency of turn | Crawford et al., 1973 |
| 516 | LEVM780103 | Second structure | Normalized frequency of reverse turn, with weights | Levitt, 1978 |
| 517 | LEVM780106 | Second structure | Normalized frequency of reverse turn, unweighted | Levitt, 1978 |
| 518 | OOBM850102 | Second structure | Optimized propensity to form reverse turn | Oobatake et al., 1985 |
| 519 | PALJ810105 | Second structure | Normalized frequency of turn from LG | Palau et al., 1981 |
| 520 | PALJ810106 | Second structure | Normalized frequency of turn from CF | Palau et al., 1981 |
| 521 | PALJ810113 | Second structure | Normalized frequency of turn in all-alpha class | Palau et al., 1981 |
| 522 | PALJ810114 | Second structure | Normalized frequency of turn in all-beta class | Palau et al., 1981 |
| 523 | PALJ810115 | Second structure | Normalized frequency of turn in alpha+beta class | Palau et al., 1981 |
| 524 | PALJ810116 | Second structure | Normalized frequency of turn in alpha/beta class | Palau et al., 1981 |
| 525 | PRAM900104 | Second structure | Relative frequency in reverse-turn | Prabhakaran, 1990 |
| 526 | ROBB760108 | Second structure | Information measure for turn | Robson-Suzuki, 1976 |
| 527 | ROBB760109 | Second structure | Information measure for N-terminal turn | Robson-Suzuki, 1976 |
| 528 | ROBB760110 | Second structure | Information measure for middle turn | Robson-Suzuki, 1976 |
| 529 | ROBB760111 | Second structure | Information measure for C-terminal turn | Robson-Suzuki, 1976 |
| 530 | SUEM840101 | Second structure | Zimm-Bragg parameter s at 20 C | Sueki et al., 1984 |
| 531 | SUEM840102 | Second structure | Zimm-Bragg parameter sigma x 1.00E+04 | Sueki et al., 1984 |
| 532 | AA number | Dynamic characteristic | Amino acid number |  |
| 533 | CC | Dynamic characteristic | Current composition of amino acids in a protein |  |
| 534 | FC | Dynamic characteristic | Future composition of amino acids in a protein | Wu-Yan, 2008 |
| 535 | DP | Dynamic characteristic | Distribution probability of amino acids in a protein | Wu-Yan, 2008 |

The constant characteristics 1–531 of amino acids can be found in AAIndex (http://www.genome.jp/aaindex/). CC is calculated with the number of amino acids of certain type divided by the total number of amino acids in a protein. FC can be calculated online (http://www.nerc-nfb.ac.cn/calculation/fc.htm). DP can be calculated online (http://www.nerc-nfb.ac.cn/calculation/dp.htm).

Table S3. Amino acids and their translated amino acids

| Amino acid | Mutated amino acids with their translation probability |
| --- | --- |
| A | 12/36A+2/36D+2/36E+4/36G+4/36P+4/36S+4/36T+4/36V |
| R | 18/54R+2/54C+2/54Q+6/54G+2/54H+1/54I+4/54L+2/54K+1/54M+4/54P+6/54S+2/54T+2/54W+2/54STOP |
| N | 2/18N+2/18D+2/18H+2/18I+4/18K+2/18S+2/18T+2/18Y |
| D | 2/18A+2/18N+2/18D+4/18E+2/18G+2/18H+2/18Y+2/18V |
| C | 2/18R+2/18C+2/18G+2/18F+4/18S+2/18W+2/18Y+2/18STOP |
| E | 2/18A+4/18D+2/18E+2/18Q+2/18G+2/18K+2/18V+2/18STOP |
| Q | 2/18R+2/18E+2/18Q+4/18H+2/18L+2/18K+2/18P+2/18STOP |
| G | 4/36A+6/36R+2/36D+2/36C+2/36E+12/36G+2/36S+1/36W+4/36V+1/36STOP |
| H | 2/18R+2/18N+2/18D+4/18Q+2/18H+2/18L+2/18P+2/18Y |
| I | 1/27R+2/27N+6/27I+4/27L+1/27K+3/27M+2/27F+2/27S+3/27T+3/27V |
| L | 4/54R+2/54Q+2/54H+4/54I+18/54L+2/54M+6/54F+4/54P+2/54S+1/54W+6/54V+3/54STOP |
| K | 2/18R+4/18N+2/18E+2/18Q+1/18I+2/18K+1/18M+2/18T+2/18STOP |
| M | 1/9R+3/9I+2/9L+1/9K+1/9T+1/9V |
| F | 2/18C+2/18I+6/18L+2/18F+2/18S+2/18Y+2/18V |
| P | 4/36A+4/36R+2/36Q+2/36H+4/36L+12/36P+4/36S+4/36T |
| S | 4/54A+6/54R+2/54N+4/54C+2/54G+2/54I+2/54L+2/54F+4/54P+14/54S+6/54T+1/54W+2/54Y+3/54STOP |
| T | 4/36A+2/36R+2/36N+3/36I+2/36K+1/36M+4/36P+6/36S+12/36T |
| W | 2/9R+2/9C+1/9G+1/9L+1/9S+2/9STOP |
| Y | 2/18N+2/18D+2/18C+2/18H+2/18F+2/18S+2/18Y+4/18STOP |
| V | 4/36A+2/36D+2/36E+4/36G+3/36I+6/36L+1/36M+2/36F+12/36V |
| STOP | 2/27R+1/27C+2/27E+2/27Q+1/27G+3/27L+2/27K+3/27S+2/27W+4/27Y+4/27STOP |
